# Supplementary material for: RNAAgeCalc: A multi-tissue transcriptional age calculator
Source: PLoS One. 2020 Aug 4;15(8):e0237006. doi: 10.1371/journal.pone.0237006 (PMC7402472; doi:10.1371/journal.pone.0237006)
Supplement: S1 Table — (PDF) [file pone.0237006.s001.pdf]

S1 Table: Summary of GTEx dataset.

tissue	sample size	gender	American Indian or Alaska Native	Asian	Black or African American	White
Adipose Tissue	617	female	0	2	35	193
		male	1	7	49	330
Adrenal Gland	158	female	0	0	9	60
		male	1	2	13	73
Bladder	11	female	0	0	0	5
		male	0	0	1	5
Blood	593	female	0	2	29	185
		male	2	1	58	316
Blood Vessel	746	female	0	4	35	244
		male	1	9	62	391
Brain	1402	female	0	11	36	395
		male	2	0	97	861
Breast	217	female	0	2	14	76
		male	0	3	16	106
Cervix Uteri	11	female	0	0	3	8
		male	0	0	0	0
Colon	374	female	0	1	18	135
		male	0	3	25	192
Esophagus	785	female	0	4	34	251
		male	3	7	63	423
Fallopian Tube	7	female	0	0	2	5
		male	0	0	0	0
Heart	487	female	0	1	21	145
		male	2	6	32	280
Kidney	36	female	0	0	1	7
		male	0	0	7	21
Liver	136	female	0	2	4	39
		male	0	1	12	78
Lung	372	female	0	1	15	112
		male	1	3	24	216
Muscle	473	female	0	2	22	149
		male	1	4	44	251
Nerve	333	female	0	2	19	96
		male	0	4	32	180
Ovary	108	female	0	2	18	88
		male	0	0	0	0
Pancreas	196	female	0	1	13	66
		male	1	3	17	95
Pituitary	123	female	0	1	4	31
		male	0	0	6	81
Prostate	118	female	0	0	0	0
		male	1	2	14	101
Salivary Gland	70	female	0	0	6	18
		male	0	0	5	41
Skin	969	female	0	3	49	292
		male	2	7	86	530
Small Intestine	103	female	0	0	5	35
		male	0	1	9	53
Spleen	118	female	0	0	9	40
		male	1	1	12	55
Stomach	202	female	0	0	13	74
		male	1	2	17	95
Testis	201	female	0	0	0	0
		male	2	2	25	172
Thyroid	360	female	0	2	17	113
		male	1	4	29	194
Uterus	90	female	0	2	16	72
		male	0	0	0	0
Vagina	97	female	0	1	14	82
		male	0	0	0	0
