# Supplementary material for: RNAAgeCalc: A multi-tissue transcriptional age calculator
Source: PLoS One. 2020 Aug 4;15(8):e0237006. doi: 10.1371/journal.pone.0237006 (PMC7402472; doi:10.1371/journal.pone.0237006)
Supplement: S2 Table — (PDF) [file pone.0237006.s002.pdf]

S2 Table: Summary of differential expression analysis on each tissue.

	total genes	FDR 0.01	FDR 0.05	FDR 0.1
adipose tissue	16086	6970	8821	9888
adrenal gland	16056	818	2369	3701
blood	14438	5015	6955	8111
blood vessel	15516	7455	8987	9836
brain	17041	8354	10090	11015
breast	16640	505	1815	2836
colon	16743	9789	11582	12444
esophagus	16388	5290	7128	8152
heart	15835	6603	8580	9686
liver	15313	932	2415	3649
lung	16740	6569	8623	9772
muscle	14460	4234	6056	7130
nerve	16219	3960	6262	7561
ovary	15879	960	2074	3058
pancreas	15350	44	182	379
pituitary	17047	154	327	530
prostate	16950	1644	3617	5047
salivary gland	16631	145	613	1271
skin	16463	2128	3542	4592
small intestine	16980	1	4	16
spleen	16222	516	1849	3042
stomach	16313	152	425	743
testis	19560	1254	2471	3476
thyroid	16517	3009	5066	6283
uterus	15971	3894	5968	7232
vagina	16644	157	651	1515
