# Supplementary material for: RNAAgeCalc: A multi-tissue transcriptional age calculator
Source: PLoS One. 2020 Aug 4;15(8):e0237006. doi: 10.1371/journal.pone.0237006 (PMC7402472; doi:10.1371/journal.pone.0237006)
Supplement: S4 Table — (PDF) [file pone.0237006.s004.pdf]

S4 Table: Summary of candidate feature sets.

candidate feature set	number of genes	type
DESeq2	1,000	tissue-specific
Pearson	1,000	tissue-specific
Deviance	2,000-7,000	tissue-specific
Peters [1]	1,497	tissue-specific (blood)
all	14,000-19,000	tissue-specific
GTExAge	1,616	across-tissue
de Magalhaes [2]	73	across-tissue
GenAge [3]	307	across-tissue

References

[1] Peters MJ, Joehanes R, Pilling LC, Schurmann C, Conneely KN, Powell J, et al. The transcriptional landscape of age in human peripheral blood. Nature communications. 2015;6:8570.

[2] De Magalhães JP, Curado J, Church GM. Meta-analysis of age-related gene expression profiles identifies common signatures of aging. Bioinformatics. 2009;25(7):875–881.

[3] Tacutu R, Thornton D, Johnson E, Budovsky A, Barardo D, Craig T, et al. Human Ageing Genomic Resources: new and updated databases. Nucleic acids research. 2017;46(D1):D1083–D1090.
