# Supplementary material for: RNAAgeCalc: A multi-tissue transcriptional age calculator
Source: PLoS One. 2020 Aug 4;15(8):e0237006. doi: 10.1371/journal.pone.0237006 (PMC7402472; doi:10.1371/journal.pone.0237006)
Supplement: S10 Table — (PDF) [file pone.0237006.s010.pdf]

S10 Table: Overlap between tissue-specific genes in GTEx and prior aging candidate genes.

prior signature	tissue	original number of genes	number of genes in GTEx	number of genes significant in GTEx*
Welle et al. [1]	muscle	479**	258	113
Lu et al. [2]	brain	463	313	232
Glass et al. [3]	adipose tissue	188	166	100
Glass et al. [3]	skin	1,672	1,334	410
Peters et al. [4]	blood	1,497	1,404	714

*Genes were considered significant if p-value was less than 0.05.

**These 479 genes corresponded to the 718 probes reported in Welle et al.

References

[1] Welle S, Brooks AI, Delehanty JM, Needler N, Thornton CA. Gene expression profile of aging in human muscle. Physiological genomics. 2003;14(2):149–159.

[2] Lu T, Pan Y, Kao SY, Li C, Kohane I, Chan J, et al. Gene regulation and DNA damage in the ageing human brain. Nature. 2004;429(6994):883.

[3] Glass D, Viñuela A, Davies MN, Ramasamy A, Parts L, Knowles D, et al. Gene expression changes with age in skin, adipose tissue, blood and brain. Genome biology. 2013;14(7):R75.

[4] Peters MJ, Joehanes R, Pilling LC, Schurmann C, Conneely KN, Powell J, et al. The transcriptional landscape of age in human peripheral blood. Nature communications. 2015;6:8570.
