# Supplementary material for: RNAAgeCalc: A multi-tissue transcriptional age calculator
Source: PLoS One. 2020 Aug 4;15(8):e0237006. doi: 10.1371/journal.pone.0237006 (PMC7402472; doi:10.1371/journal.pone.0237006)
Supplement: S11 Table — (PDF) [file pone.0237006.s011.pdf]

S11 Table: Comparison of the sign of fold change between GTEx and prior aging candidate genes.

Lu (brain) [1] p = 2.18e-39			Glass (adipose tissue) [2] p = 0.0179		
GTEx	+	-	GTEx	+	-
+	170	12	+	49	37
-	31	99	-	28	46
Glass (skin) [2] p = 5.71e-53			Peters (whole blood) [3] p = 8.96e-14		
GTEx	+	-	GTEx	+	-
+	446	211	+	328	303
-	156	456	-	237	502

References

[1] Lu T, Pan Y, Kao SY, Li C, Kohane I, Chan J, et al. Gene regulation and DNA damage in the ageing human brain. Nature. 2004;429(6994):883.

[2] Glass D, Viñuela A, Davies MN, Ramasamy A, Parts L, Knowles D, et al. Gene expression changes with age in skin, adipose tissue, blood and brain. Genome biology. 2013;14(7):R75.

[3] Peters MJ, Joehanes R, Pilling LC, Schurmann C, Conneely KN, Powell J, et al. The transcriptional landscape of age in human peripheral blood. Nature communications. 2015;6:8570.
