# Supplementary material for: RNAAgeCalc: A multi-tissue transcriptional age calculator
Source: PLoS One. 2020 Aug 4;15(8):e0237006. doi: 10.1371/journal.pone.0237006 (PMC7402472; doi:10.1371/journal.pone.0237006)
Supplement: S12 Table — (PDF) [file pone.0237006.s012.pdf]

S12 Table: Comparison of GTEx aging genes versus prior candidate genes on within-tissue prediction.

tissue	signature	Pearson_cor	Spearman_cor	median_error	mean_error	RMSE
adipose_tissue	DESeq2	0.73	0.69	5.35	6.65	8.56
	Pearson	0.72	0.68	5.72	6.87	8.75
	Glass [1]	0.57	0.52	6.91	8.22	10.31
blood	DESeq2	0.38	0.38	8.38	9.85	12.21
	Pearson	0.35	0.35	8.31	9.85	12.26
	Peters [2]	0.44	0.44	7.86	9.43	11.78
brain	DESeq2	0.82	0.75	3.65	4.56	6.01
	Pearson	0.81	0.73	3.88	4.81	6.24
	Lu [3]	0.73	0.63	4.55	5.61	7.25
muscle	DESeq2	0.75	0.68	5.60	6.79	8.67
	Pearson	0.73	0.65	5.72	7.03	8.99
	Welle [4]	0.58	0.55	7.28	8.58	10.74
skin	DESeq2	0.56	0.56	6.66	8.19	10.48
	Pearson	0.58	0.57	6.51	8.00	10.28
	Glass [1]	0.54	0.52	6.91	8.35	10.64
skin_fibroblast	DESeq2	0.42	0.42	7.43	9.30	11.93
	Pearson	0.43	0.43	7.75	9.37	11.92
	Glass [1]	0.25	0.28	7.69	9.66	12.52
skin_nonfibroblast	DESeq2	0.66	0.65	5.96	7.23	9.26
	Pearson	0.63	0.63	6.41	7.49	9.58
	Glass [1]	0.59	0.59	6.42	7.74	9.92

References

[1] Glass D, Viñuela A, Davies MN, Ramasamy A, Parts L, Knowles D, et al. Gene expression changes with age in skin, adipose tissue, blood and brain. Genome biology. 2013;14(7):R75.

[2] Peters MJ, Joehanes R, Pilling LC, Schurmann C, Conneely KN, Powell J, et al. The transcriptional landscape of age in human peripheral blood. Nature communications. 2015;6:8570.

[3] Lu T, Pan Y, Kao SY, Li C, Kohane I, Chan J, et al. Gene regulation and DNA damage in the ageing human brain. Nature. 2004;429(6994):883.

[4] Welle S, Brooks AI, Delehanty JM, Needler N, Thornton CA. Gene expression profile of aging in human muscle. Physiological genomics. 2003;14(2):149–159.
