# Supplementary material for: RNAAgeCalc: A multi-tissue transcriptional age calculator
Source: PLoS One. 2020 Aug 4;15(8):e0237006. doi: 10.1371/journal.pone.0237006 (PMC7402472; doi:10.1371/journal.pone.0237006)
Supplement: S13 Table — (PDF) [file pone.0237006.s013.pdf]

S13 Table: Overlap between GTExAge genes and prior aging candidate genes.

prior signature	original number of genes	number of genes in GTEx	number of significant genes* (pos. related)	number of significant genes (neg. related)
deMagalhaes [1]	73	64	12 (18.75%)	6 (9.38%)
GenAge [2]	307	302	40 (13.25%)	42 (13.91%)
Horvath [3]	344**	267	27 (10.11%)	22 (8.24%)

*Genes were considered significant if p-value is less than 0.05 in the binomial test.

**These 344 genes corresponded to the 353 probes reported in Horvath et al.

References

[1] De Magalhães JP, Curado J, Church GM. Meta-analysis of age-related gene expression profiles identifies common signatures of aging. Bioinformatics. 2009;25(7):875–881.

[2] Tacutu R, Thornton D, Johnson E, Budovsky A, Barardo D, Craig T, et al. Human Ageing Genomic Resources: new and updated databases. Nucleic acids research. 2017;46(D1):D1083–D1090.

[3] Horvath S. DNA methylation age of human tissues and cell types. Genome biology. 2013;14(10):3156.
