# Supplementary material for: RNAAgeCalc: A multi-tissue transcriptional age calculator
Source: PLoS One. 2020 Aug 4;15(8):e0237006. doi: 10.1371/journal.pone.0237006 (PMC7402472; doi:10.1371/journal.pone.0237006)
Supplement: S14 Table — (PDF) [file pone.0237006.s014.pdf]

S14 Table: Correlation between age acceleration residual and mutation burden (based on DESeq2 and GTExAge genes).

	transcriptional age acceleration (based on DESeq2 genes)					
	Pearson_r	Pearson_pv	Pearson_pvadj	Spearman_r	Spearman_pv	Spearman_pvadj
ACC	-3.32E-01	2.78E-03	1.39E-02	-3.66E-01	9.06E-04	4.53E-03
BRCA	-1.12E-03	9.77E-01	9.77E-01	1.84E-02	6.34E-01	6.79E-01
GBMLGG	-2.11E-01	6.53E-07	4.90E-06	-1.59E-01	1.85E-04	1.39E-03
COADREAD	1.13E-01	2.67E-02	8.02E-02	2.88E-02	5.75E-01	6.63E-01
ESCA	-5.23E-03	9.44E-01	9.77E-01	-5.73E-02	4.42E-01	6.15E-01
LIHC	-8.02E-02	1.36E-01	2.90E-01	-6.59E-02	2.20E-01	5.49E-01
LUAD	-1.81E-01	3.67E-07	4.90E-06	-1.82E-01	3.12E-07	4.68E-06
OV	-7.41E-01	2.22E-02	8.02E-02	-7.67E-01	2.14E-02	8.02E-02
PAAD	-6.28E-02	4.26E-01	6.38E-01	-6.60E-02	4.02E-01	6.15E-01
PRAD	5.15E-03	9.10E-01	9.77E-01	5.00E-02	2.74E-01	5.88E-01
SKCM (tumor)	1.38E-01	1.65E-01	3.09E-01	9.43E-02	3.43E-01	6.15E-01
STAD	2.84E-02	5.91E-01	8.06E-01	3.98E-02	4.51E-01	6.15E-01
TGCT	1.01E-01	2.53E-01	4.21E-01	6.07E-02	4.94E-01	6.18E-01
THCA	-7.62E-02	9.53E-02	2.38E-01	-7.37E-02	1.07E-01	3.20E-01
SKCM (metastatic)	7.55E-03	8.86E-01	9.77E-01	1.98E-02	7.08E-01	7.08E-01
all tissues	-2.97E-02	3.45E-02		-3.71E-02	8.22E-03	

	transcriptional age acceleration (based on GTExAge genes)					
	Pearson_r	Pearson_pv	Pearson_pvadj	Spearman_r	Spearman_pv	Spearman_pvadj
ACC	-2.17E-01	5.48E-02	1.41E-01	-2.22E-01	4.92E-02	1.23E-01
BRCA	-3.13E-02	4.16E-01	5.67E-01	-3.84E-02	3.19E-01	4.35E-01
GBMLGG	-2.24E-01	1.17E-07	8.75E-07	-2.03E-01	1.67E-06	1.25E-05
COADREAD	-6.29E-02	2.20E-01	3.78E-01	2.68E-03	9.58E-01	9.58E-01
ESCA	-2.32E-02	7.56E-01	8.10E-01	-9.08E-02	2.23E-01	3.45E-01
LIHC	5.19E-03	9.23E-01	9.23E-01	1.70E-02	7.52E-01	8.05E-01
LUAD	-2.42E-01	7.99E-12	1.20E-10	-2.36E-01	2.68E-11	4.02E-10
OV	-4.37E-01	2.40E-01	3.78E-01	-4.50E-01	2.30E-01	3.45E-01
PAAD	2.97E-01	1.16E-04	5.82E-04	2.85E-01	2.30E-04	1.15E-03
PRAD	7.26E-02	1.13E-01	2.41E-01	7.88E-02	8.49E-02	1.82E-01
SKCM (tumor)	1.14E-01	2.52E-01	3.78E-01	7.55E-02	4.48E-01	5.60E-01
STAD	3.19E-02	5.46E-01	6.82E-01	7.58E-02	1.51E-01	2.83E-01
TGCT	-2.36E-01	7.21E-03	2.70E-02	-2.15E-01	1.42E-02	4.27E-02
THCA	8.70E-02	5.64E-02	1.41E-01	1.16E-01	1.08E-02	4.05E-02
SKCM (metastatic)	1.68E-02	7.50E-01	8.10E-01	2.40E-02	6.50E-01	7.50E-01
all tissues	-4.03E-02	4.08E-03		-4.66E-02	9.01E-04	
