# Supplementary material for: RNAAgeCalc: A multi-tissue transcriptional age calculator
Source: PLoS One. 2020 Aug 4;15(8):e0237006. doi: 10.1371/journal.pone.0237006 (PMC7402472; doi:10.1371/journal.pone.0237006)
Supplement: S15 Table — (PDF) [file pone.0237006.s015.pdf]

S15 Table: Coefficient and p-value of age acceleration residual from Cox regression (based on DESeq2 and GTExAge genes).

	transcriptional age acceleration (based on DESeq2 genes)					
	Coef_Mod0a	PV_Mod0a	PVadj_Mod0a	Coef_Mod1a	PV_Mod1a	PVadj_Mod1a
ACC	-5.90E-02	1.35E-04	2.02E-03	-4.48E-02	1.34E-02	1.45E-01
BRCA	6.01E-03	2.08E-01	4.47E-01	3.88E-03	4.40E-01	7.32E-01
GBMLGG	-1.31E-02	4.21E-02	2.11E-01	-1.31E-02	4.21E-02	2.11E-01
COADREAD	7.08E-03	2.99E-01	5.60E-01	1.59E-03	8.28E-01	8.98E-01
ESCA	1.00E-02	1.37E-01	3.98E-01	5.13E-03	4.88E-01	7.32E-01
LIHC	-8.77E-03	8.97E-02	3.36E-01	-4.17E-03	4.35E-01	7.32E-01
LUAD	8.24E-04	7.88E-01	9.21E-01	2.84E-03	3.58E-01	7.32E-01
OV	-2.93E-02	3.55E-01	5.92E-01	-2.93E-02	3.55E-01	7.32E-01
PAAD	-1.22E-02	1.59E-01	3.98E-01	-1.16E-02	1.98E-01	7.32E-01
PRAD	2.07E-02	8.51E-01	9.21E-01	2.07E-02	8.51E-01	8.98E-01
SKCM (tumor)	-1.42E-03	8.59E-01	9.21E-01	-1.89E-03	8.35E-01	8.98E-01
STAD	-1.96E-03	5.05E-01	6.89E-01	-5.01E-04	8.68E-01	8.98E-01
TGCT	-2.63E-03	9.60E-01	9.60E-01	6.70E-03	8.98E-01	8.98E-01
THCA	-1.91E-02	4.15E-01	6.23E-01	-2.54E-02	3.54E-01	7.32E-01
SKCM (metastatic)	-6.86E-03	2.23E-02	1.68E-01	-7.10E-03	1.93E-02	1.45E-01
	transcriptional age acceleration (based on GTExAge genes)					
	Coef_Mod0a	PV_Mod0a	PVadj_Mod0a	Coef_Mod1a	PV_Mod1a	PVadj_Mod1a
ACC	-1.15E-02	2.91E-01	5.46E-01	1.68E-02	1.88E-01	3.85E-01
BRCA	7.95E-04	9.06E-01	9.06E-01	1.94E-03	7.65E-01	7.65E-01
GBMLGG	-2.44E-02	5.08E-09	7.62E-08	-2.44E-02	5.08E-09	7.62E-08
COADREAD	1.58E-02	3.57E-03	1.79E-02	8.98E-03	1.47E-01	3.67E-01
ESCA	4.25E-03	4.94E-01	6.74E-01	2.91E-03	6.94E-01	7.65E-01
LIHC	3.07E-03	5.79E-01	7.00E-01	2.31E-03	6.83E-01	7.65E-01
LUAD	-6.57E-04	7.85E-01	8.41E-01	8.74E-04	7.24E-01	7.65E-01
OV	-6.73E-02	1.39E-01	3.49E-01	-6.73E-02	1.39E-01	3.67E-01
PAAD	-2.41E-03	6.06E-01	7.00E-01	-2.77E-03	5.53E-01	7.54E-01
PRAD	-1.04E-01	3.87E-01	5.80E-01	-1.04E-01	3.87E-01	5.81E-01
SKCM (tumor)	-5.99E-03	3.59E-01	5.80E-01	-6.16E-03	3.87E-01	5.81E-01
STAD	3.98E-03	1.39E-01	3.49E-01	4.46E-03	1.01E-01	3.67E-01
TGCT	5.51E-02	2.89E-01	5.46E-01	1.15E-01	2.05E-01	3.85E-01
THCA	-2.51E-02	7.01E-02	2.63E-01	-3.50E-02	3.21E-02	1.60E-01
SKCM (metastatic)	-8.00E-03	2.22E-04	1.67E-03	-8.21E-03	1.25E-04	9.39E-04
