# Supplementary material for: RNAAgeCalc: A multi-tissue transcriptional age calculator
Source: PLoS One. 2020 Aug 4;15(8):e0237006. doi: 10.1371/journal.pone.0237006 (PMC7402472; doi:10.1371/journal.pone.0237006)
Supplement: S16 Table — (PDF) [file pone.0237006.s016.pdf]

S16 Table: Coefficient and p-value of age acceleration residual versus stage from linear model (based on DESeq2 and GTExAge genes).

	transcriptional age acceleration (based on DESeq2 genes)					
	Coef_Mod0b	PV_Mod0b	PVadj_Mod0b	Coef_Mod1b	PV_Mod1b	PVadj_Mod1b
ACC	-6.3156	0.0027	0.0160	-6.2440	0.0031	0.0132
BRCA	0.6702	0.0224	0.0673	0.6690	0.0221	0.0662
COADREAD	0.2619	0.2242	0.3363	0.2661	0.2168	0.3252
ESCA	1.9957	0.0122	0.0490	2.4887	0.0012	0.0132
LIHC	-1.8313	0.0027	0.0160	-1.7734	0.0033	0.0132
LUAD	-0.2908	0.3410	0.4091	-0.2251	0.4615	0.5538
PAAD	-1.3223	0.1637	0.3274	-1.3465	0.1562	0.2761
SKCM (tumor)	-1.0419	0.3247	0.4091	-0.9442	0.3763	0.5017
STAD	-0.9390	0.1923	0.3297	-1.0148	0.1611	0.2761
TGCT	0.3528	0.1241	0.2979	0.3475	0.1326	0.2761
THCA	-0.1618	0.7349	0.8018	-0.1312	0.7838	0.8551
SKCM (metastatic)	-0.0244	0.9505	0.9505	-0.0505	0.8986	0.8986

	transcriptional age acceleration (based on GTExAge genes)					
	Coef_Mod0b	PV_Mod0b	PVadj_Mod0b	Coef_Mod1b	PV_Mod1b	PVadj_Mod1b
ACC	-6.9470	0.0083	0.0499	-6.8472	0.0106	0.0636
BRCA	0.0069	0.9768	0.9768	0.0069	0.9767	0.9767
COADREAD	0.8715	0.0006	0.0067	0.8782	0.0005	0.0056
ESCA	0.7202	0.4026	0.6837	1.4558	0.0707	0.2121
LIHC	0.4764	0.4078	0.6837	0.4692	0.4180	0.7214
LUAD	-0.3542	0.3922	0.6837	-0.2455	0.5527	0.8290
PAAD	0.7633	0.6926	0.9235	0.7990	0.6804	0.9072
SKCM (tumor)	-1.2592	0.3198	0.6837	-1.0352	0.4208	0.7214
STAD	0.1052	0.8900	0.9709	0.0863	0.9103	0.9767
TGCT	-0.5650	0.0255	0.1021	-0.5888	0.0203	0.0812
THCA	0.4354	0.4558	0.6837	0.4814	0.4079	0.7214
SKCM (metastatic)	-0.1027	0.8524	0.9709	-0.1609	0.7722	0.9267
