# Supplementary figures and images for: RNAAgeCalc: A multi-tissue transcriptional age calculator
Source: PLoS One. 2020 Aug 4;15(8):e0237006. doi: 10.1371/journal.pone.0237006 (PMC7402472; doi:10.1371/journal.pone.0237006)

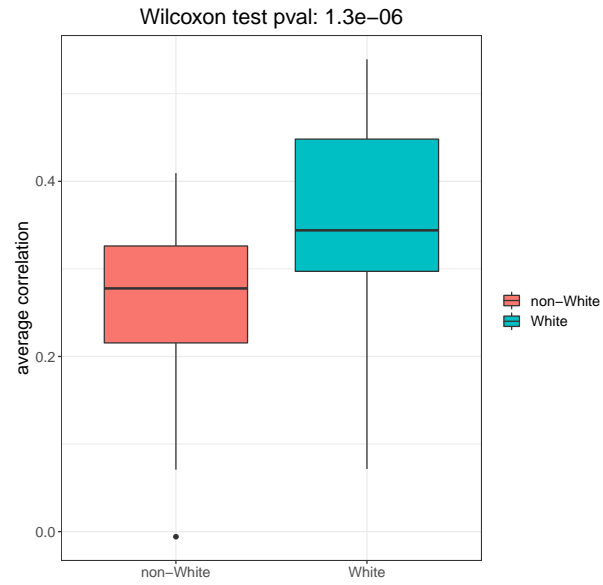

S2 Fig: Comparison of the prediction on White and non-White samples (based on GTExAge genes).

Supplement: S2 Fig — (PDF) [file pone.0237006.s019.pdf]
