# Supplementary material for: RNAAgeCalc: A multi-tissue transcriptional age calculator
Source: PLoS One. 2020 Aug 4;15(8):e0237006. doi: 10.1371/journal.pone.0237006 (PMC7402472; doi:10.1371/journal.pone.0237006)
Supplement: S3 Fig — (PDF) [file pone.0237006.s020.pdf]

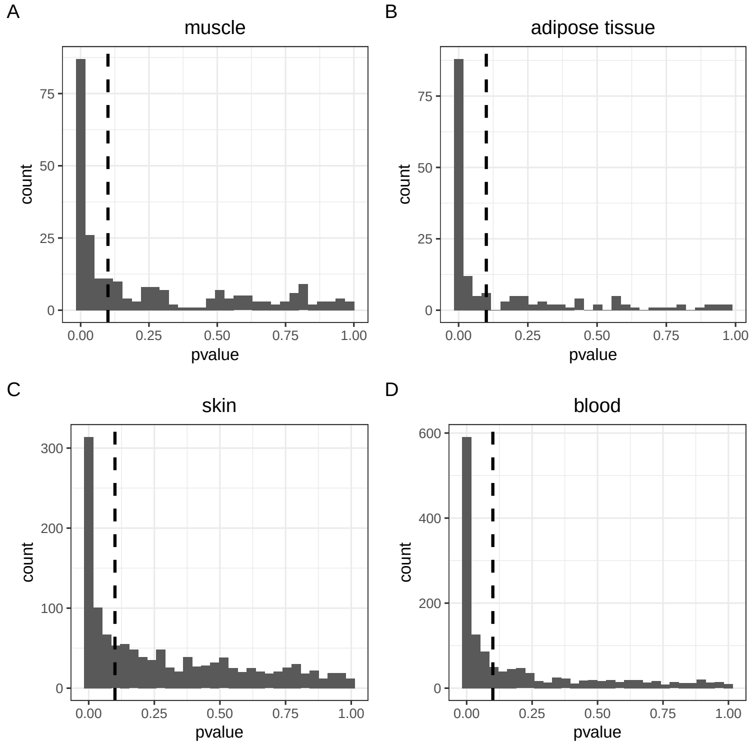

S3 Fig: Histogram of p-values of prior candidate genes in GTEx data computed from DESeq2. Dashed line corresponded to p-value 0.1.
