# Supplementary material for: RNAAgeCalc: A multi-tissue transcriptional age calculator
Source: PLoS One. 2020 Aug 4;15(8):e0237006. doi: 10.1371/journal.pone.0237006 (PMC7402472; doi:10.1371/journal.pone.0237006)
Supplement: S4 Fig — (PDF) [file pone.0237006.s021.pdf]

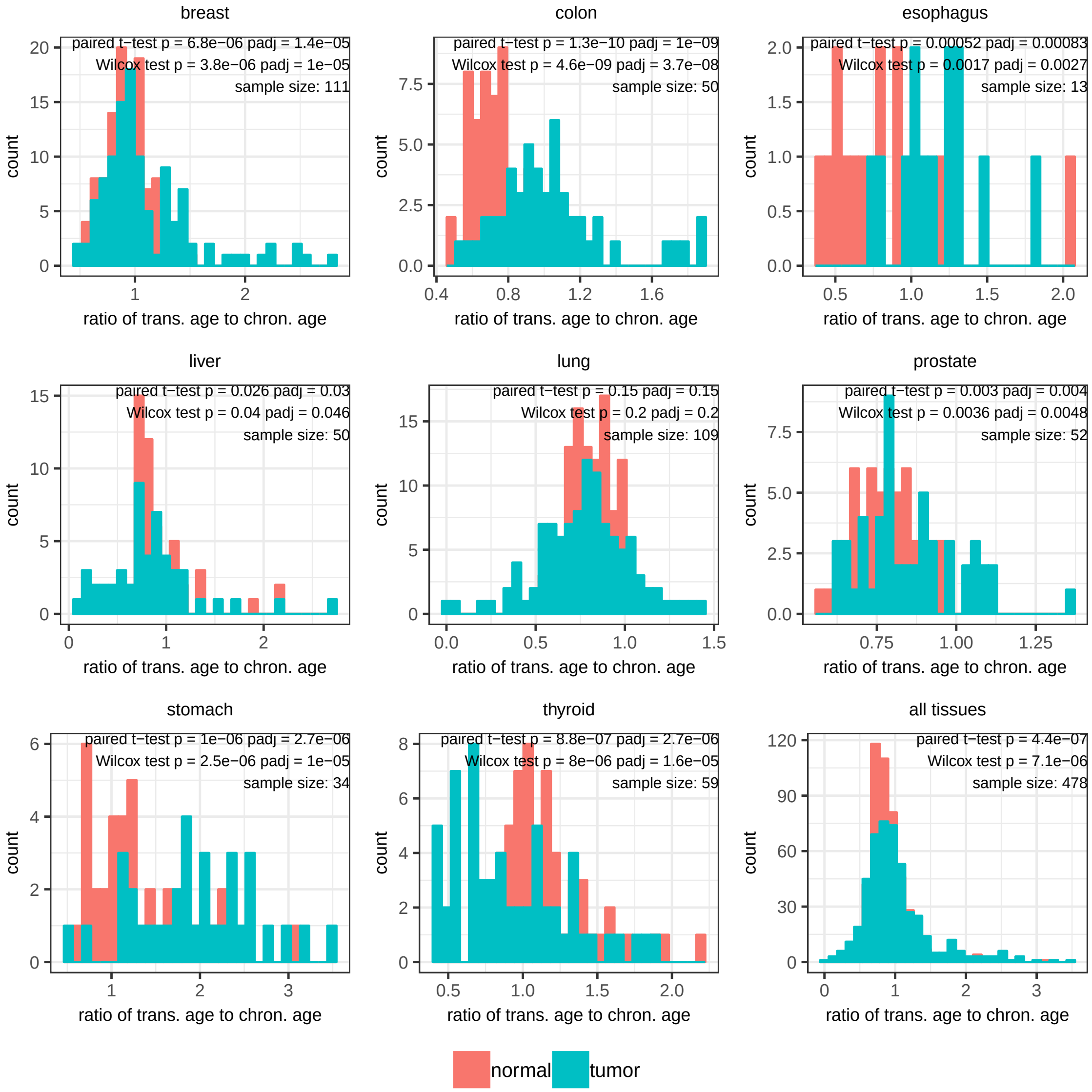

S4 Fig: Ratio of transcriptional age to chronological age on matched tumor and normal samples from TCGA.
