# Supplementary material for: RNAAgeCalc: A multi-tissue transcriptional age calculator
Source: PLoS One. 2020 Aug 4;15(8):e0237006. doi: 10.1371/journal.pone.0237006 (PMC7402472; doi:10.1371/journal.pone.0237006)
Supplement: S1 Appendix — (PDF) [file pone.0237006.s022.pdf]

Comparison of elastic net and ensemble LDA.

As discussed in the main manuscript, the only predictor constructed using RNA-Seq data was the ensemble LDA predictor based on fibroblast data [1]. To evaluate the transferability of the ensemble LDA model, we conducted an across-data prediction. The prediction evaluation consisted of two parts: (i) using each GTEx tissue as training set in refitting the ensemble LDA model, and evaluating the performance on the dermal fibroblast data of [1]; (ii) using the dermal fibroblast data of [1] as training set in refitting the ensemble LDA model, and evaluating the performance in each GTEx tissue. We retrained the ensemble LDA on the dermal fibroblast data because a subset of genes in the original ensemble LDA model was not found on the GTEx dataset. In part (i), we also considered the prediction models trained on all tissues combined and tested on the dermal fibroblast data. Two strategies were considered in the all-tissue model. In the first strategy, samples from all tissues in GTEx were combined and one prediction model was trained on all samples. In the second strategy, one prediction model was trained on each GTEx tissue and the prediction scores were averaged across all the prediction models. Both the elastic net and ensemble LDA trained on each candidate feature set in the Methods section of the main manuscript were considered with a few exceptions. When constructing ensemble LDA model on the dermal fibroblast data, DESeq signature was not considered because the read count data was not available, and therefore we were unable to carry out differential expression analysis. The all genes signature for ensemble LDA was also not considered due to the high computational cost. In the first strategy of all-tissue model, DESeq signature was not considered as the differential expression analysis on $> 9{,}000$ samples was computationally expensive.

The prediction results were provided in S6 Table. Despite some existing literatures suggesting that fibroblast was a good candidate tissue for studying age-dependent transcriptional changes [2, 3, 4, 5, 6, 7], the ensemble LDA model based on dermal fibroblast data of [8] was not generalizable to other tissues as the prediction accuracy on GTEx tissues was relatively low. This could be partially attributed to the batch effect between the different datasets. Similarly, elastic net model also yielded poor accuracy when trained on the dermal fibroblast data and tested on GTEx tissues. However, elastic net model performed better than ensemble LDA when trained on GTEx tissues and tested on dermal fibroblast data of [1]. The results in this subsection, together with the poor performance of ensemble LDA in within-tissue prediction section illustrated that ensemble LDA only worked well within the dermal fibroblast data [1] and cannot be generalized to predict transcriptional age in other datasets.

References

[1] Fleischer JG, Schulte R, Tsai HH, Tyagi S, Ibarra A, Shokhirev MN, et al. Predicting age from the transcriptome of human dermal fibroblasts. Genome biology. 2018;19(1):221.

[2] Grönniger E, Weber B, Heil O, Peters N, Stäb F, Wenck H, et al. Aging and chronic sun exposure cause distinct epigenetic changes in human skin. PLoS genetics. 2010;6(5):e1000971.

[3] Glass D, Viñuela A, Davies MN, Ramasamy A, Parts L, Knowles D, et al. Gene expression changes with age in skin, adipose tissue, blood and brain. Genome biology. 2013;14(7):R75.

[4] Tigges J, Krutmann J, Fritsche E, Haendeler J, Schaal H, Fischer JW, et al. The hallmarks of fibroblast ageing. Mechanisms of ageing and development. 2014;138:26–44.

[5] Jung M, Jin SG, Zhang X, Xiong W, Gogoshin G, Rodin AS, et al. Longitudinal epigenetic and gene expression profiles analyzed by three-component analysis reveal downregulation of genes involved in protein translation in human aging. Nucleic acids research. 2015;43(15):e100–e100.

[6] Phillip JM, Wu PH, Gilkes DM, Williams W, McGovern S, Daya J, et al. Biophysical and biomolecular determination of cellular age in humans. Nature Biomedical Engineering. 2017;1(7):0093.

[7] Kaisers W, Boukamp P, Stark HJ, Schwender H, Tigges J, Krutmann J, et al. Age, gender and UV-exposition related effects on gene expression in in vivo aged short term cultivated human dermal fibroblasts. PLoS One. 2017;12(5):e0175657.

[8] Hastie T, Tibshirani R, Narasimhan B, Chu G. impute: Imputation for microarray data; 2020. R package version 1.62.0.
