# Supplementary material for: RNAAgeCalc: A multi-tissue transcriptional age calculator
Source: PLoS One. 2020 Aug 4;15(8):e0237006. doi: 10.1371/journal.pone.0237006 (PMC7402472; doi:10.1371/journal.pone.0237006)
Supplement: S2 Appendix — (PDF) [file pone.0237006.s023.pdf]

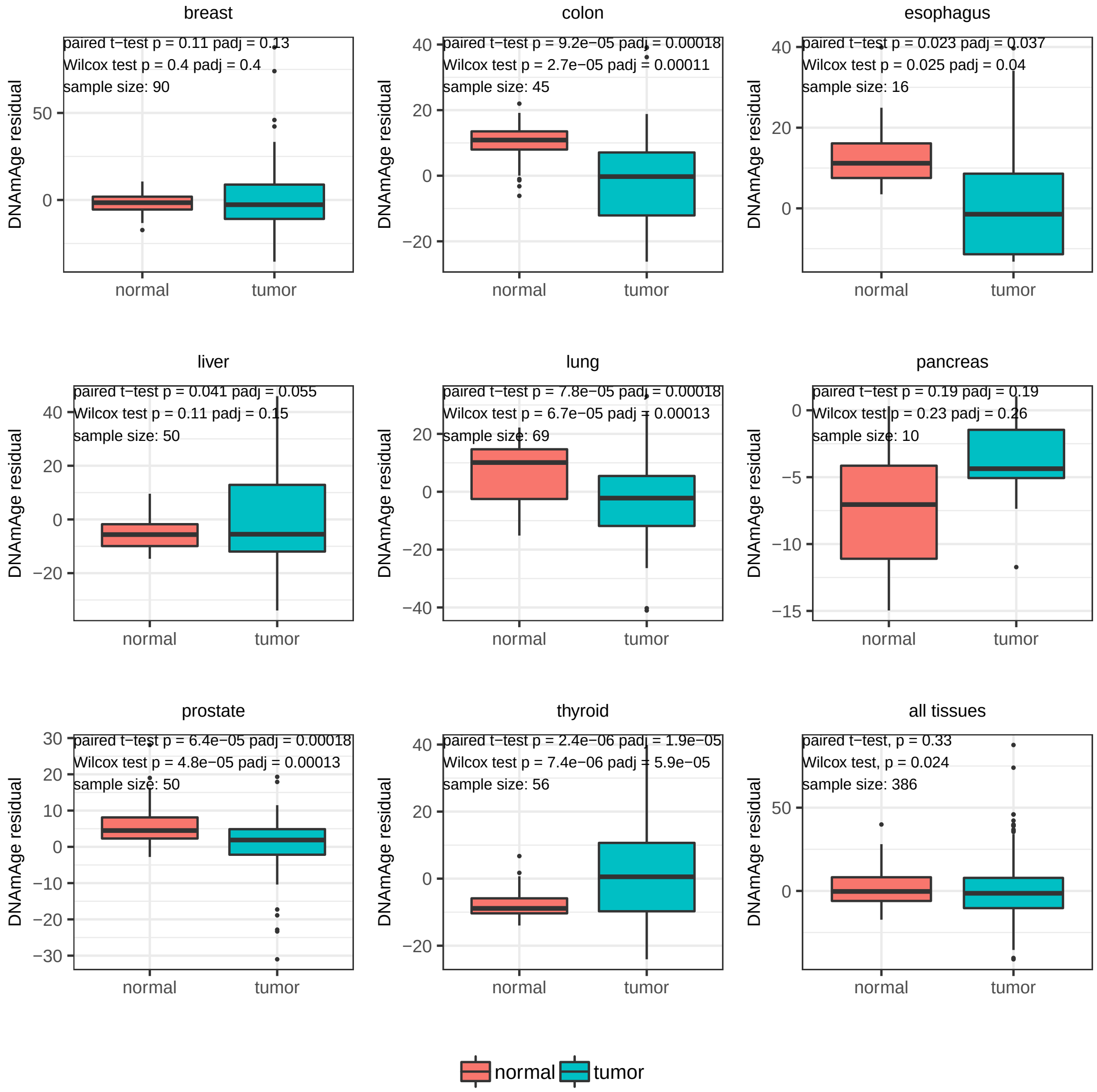

Age predictions on matched tumor and normal samples from TCGA (based on Horvath DNAm clock).

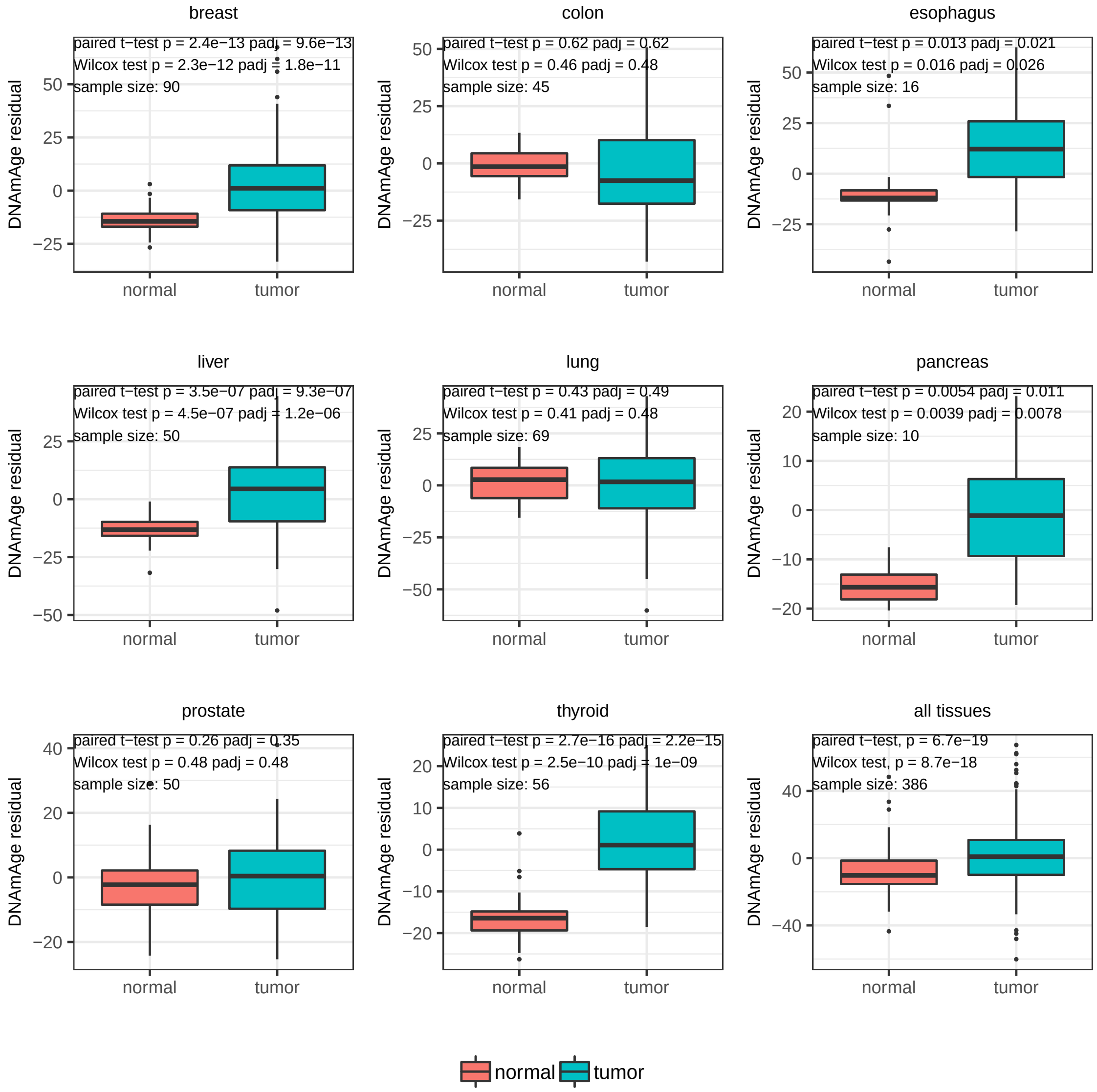

Age predictions on matched tumor and normal samples from TCGA (based on Hannum DNAm clock).

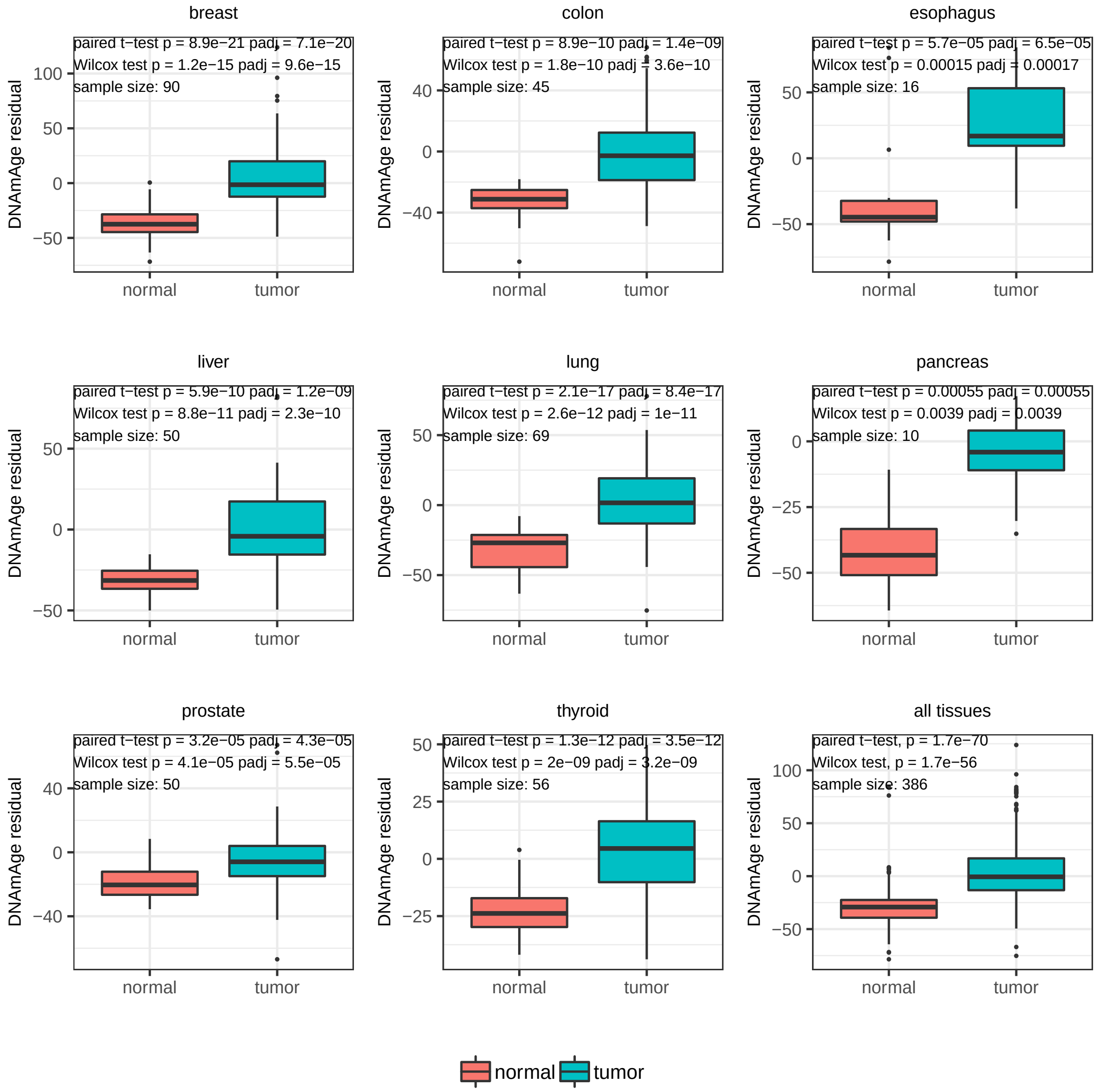

Age predictions on matched tumor and normal samples from TCGA (based on Levine DNAm clock).

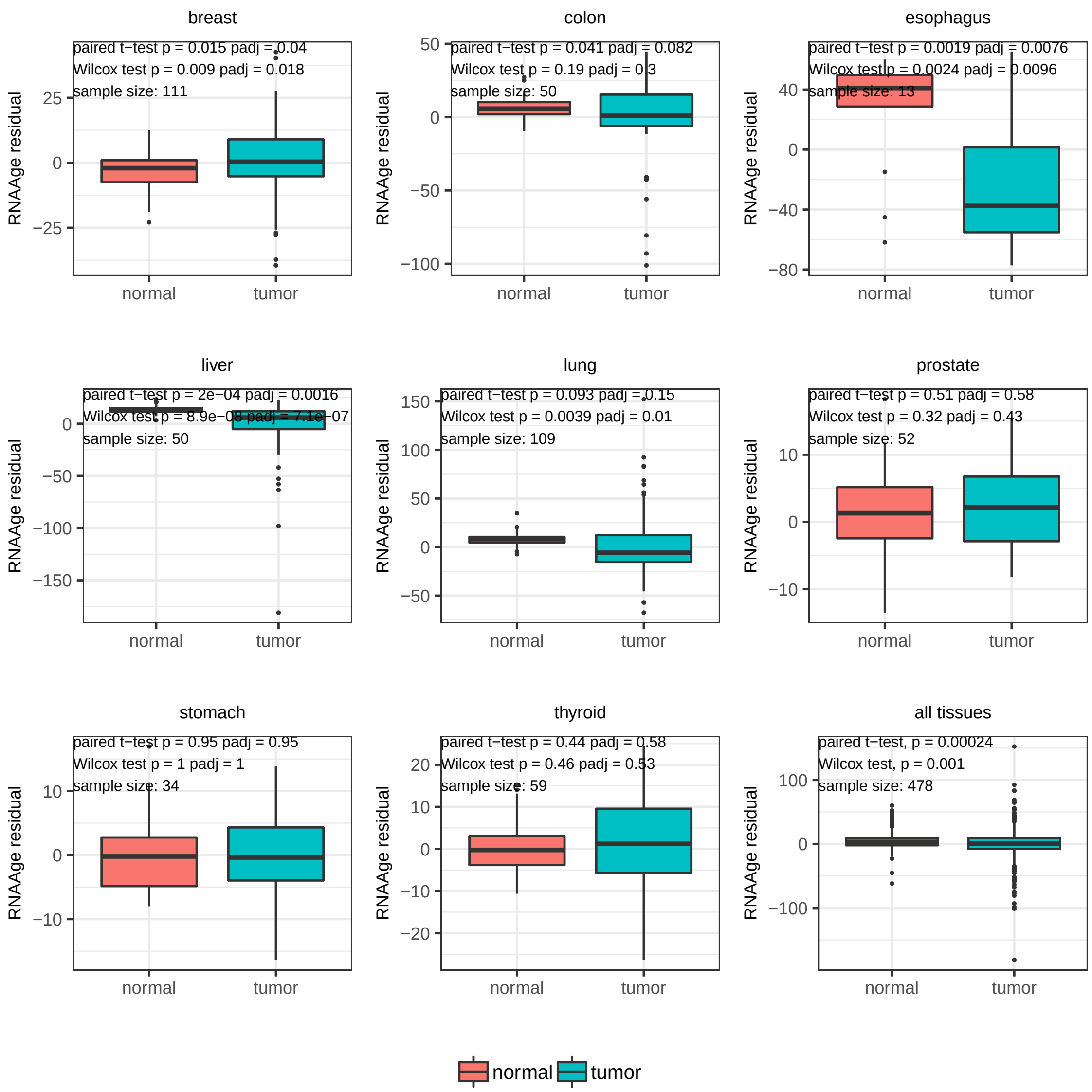

Age predictions on matched tumor and normal samples from TCGA (based on Horvath genes). Horvath genes was defined as the genes corresponding to the 353 CpGs in Horvath's clock.

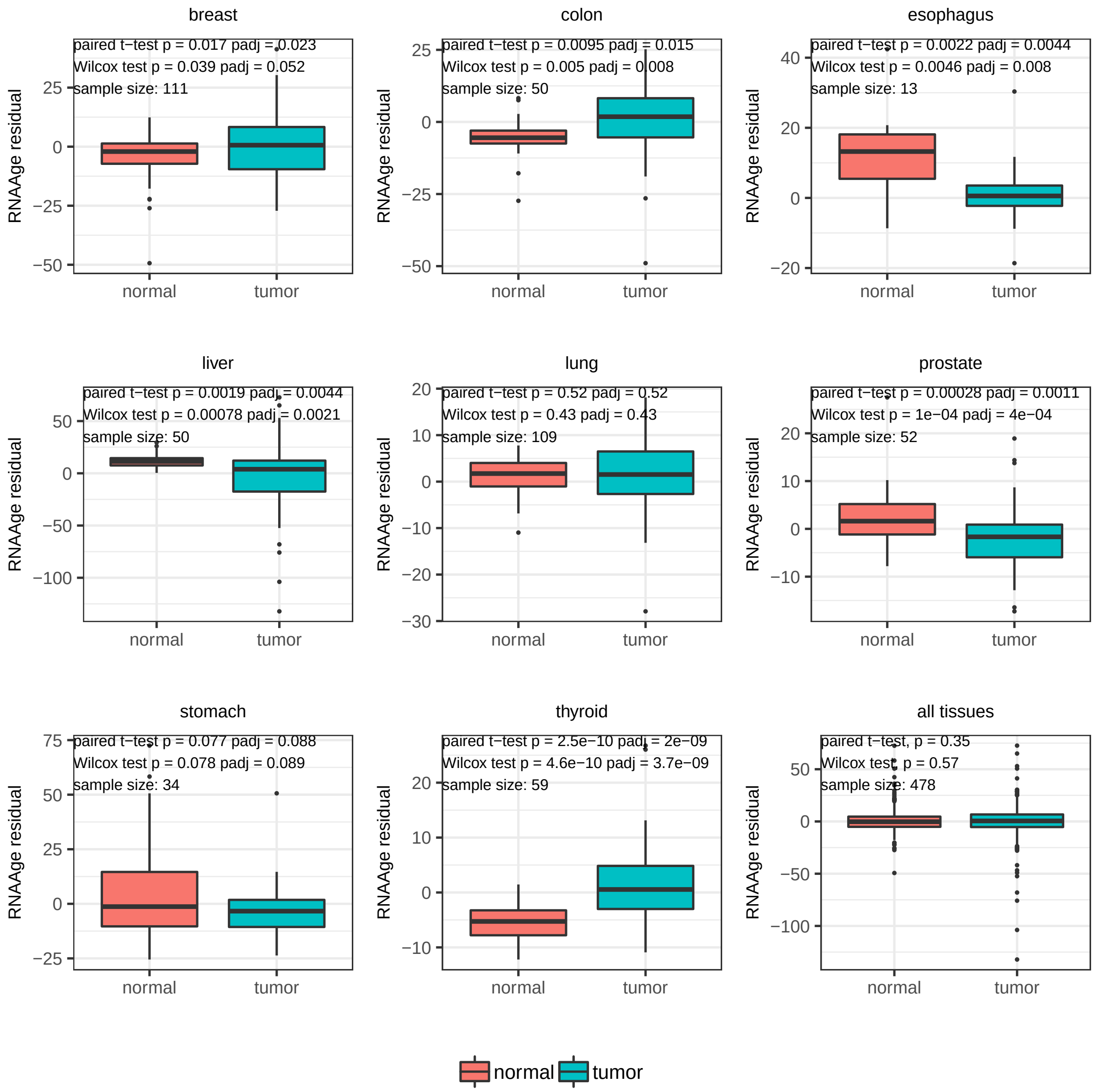

Age predictions on matched tumor and normal samples from TCGA (based on Hannum genes). Hannum genes was defined as the genes corresponding to the 71 CpGs in Hannum's clock.

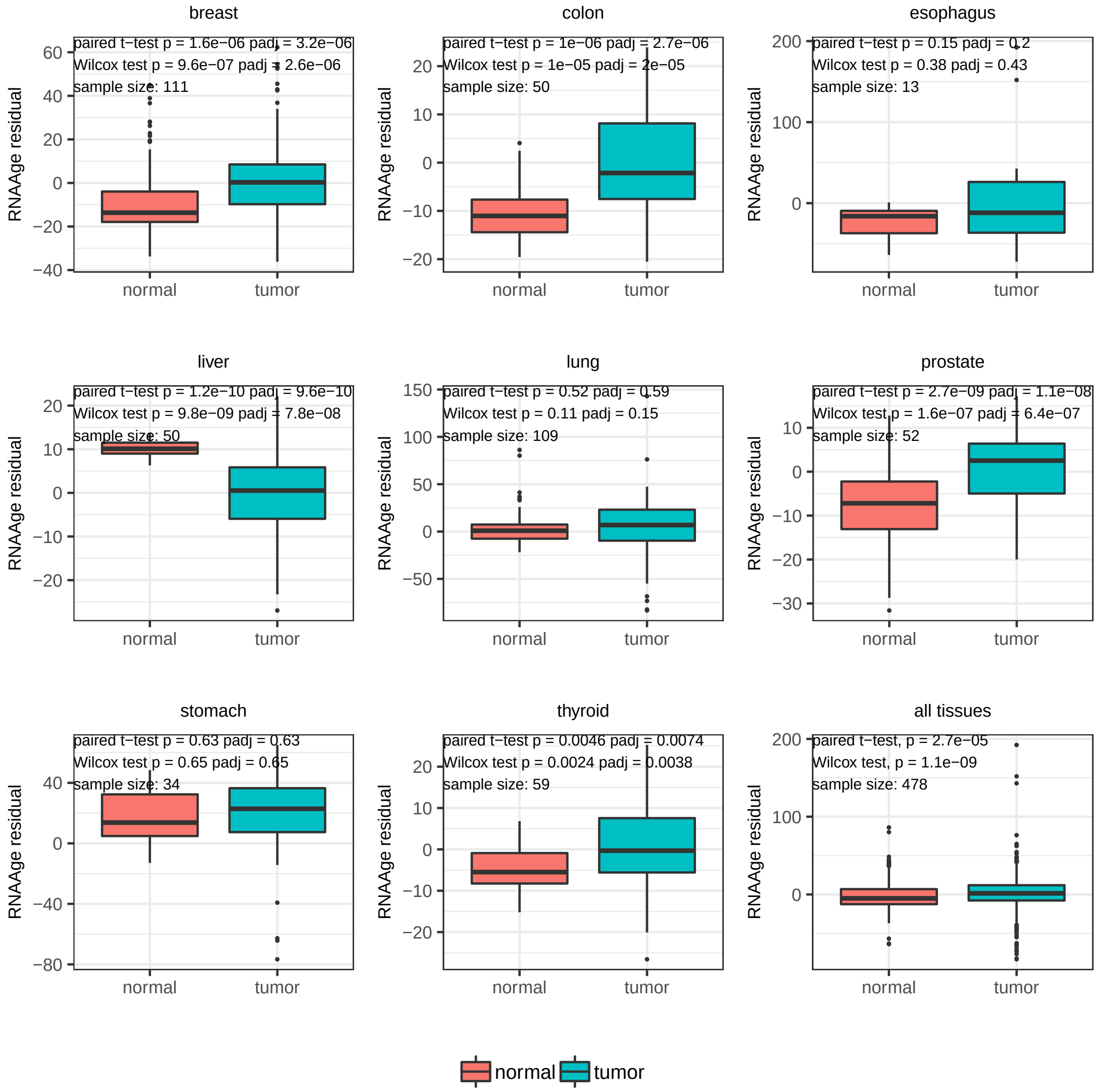

Age predictions on matched tumor and normal samples from TCGA (based on Levine genes). Levine genes was defined as the genes corresponding to the 513 CpGs in Levine's clock.
