# Supplementary material for: RNAAgeCalc: A multi-tissue transcriptional age calculator
Source: PLoS One. 2020 Aug 4;15(8):e0237006. doi: 10.1371/journal.pone.0237006 (PMC7402472; doi:10.1371/journal.pone.0237006)
Supplement: S3 Appendix — (PDF) [file pone.0237006.s024.pdf]

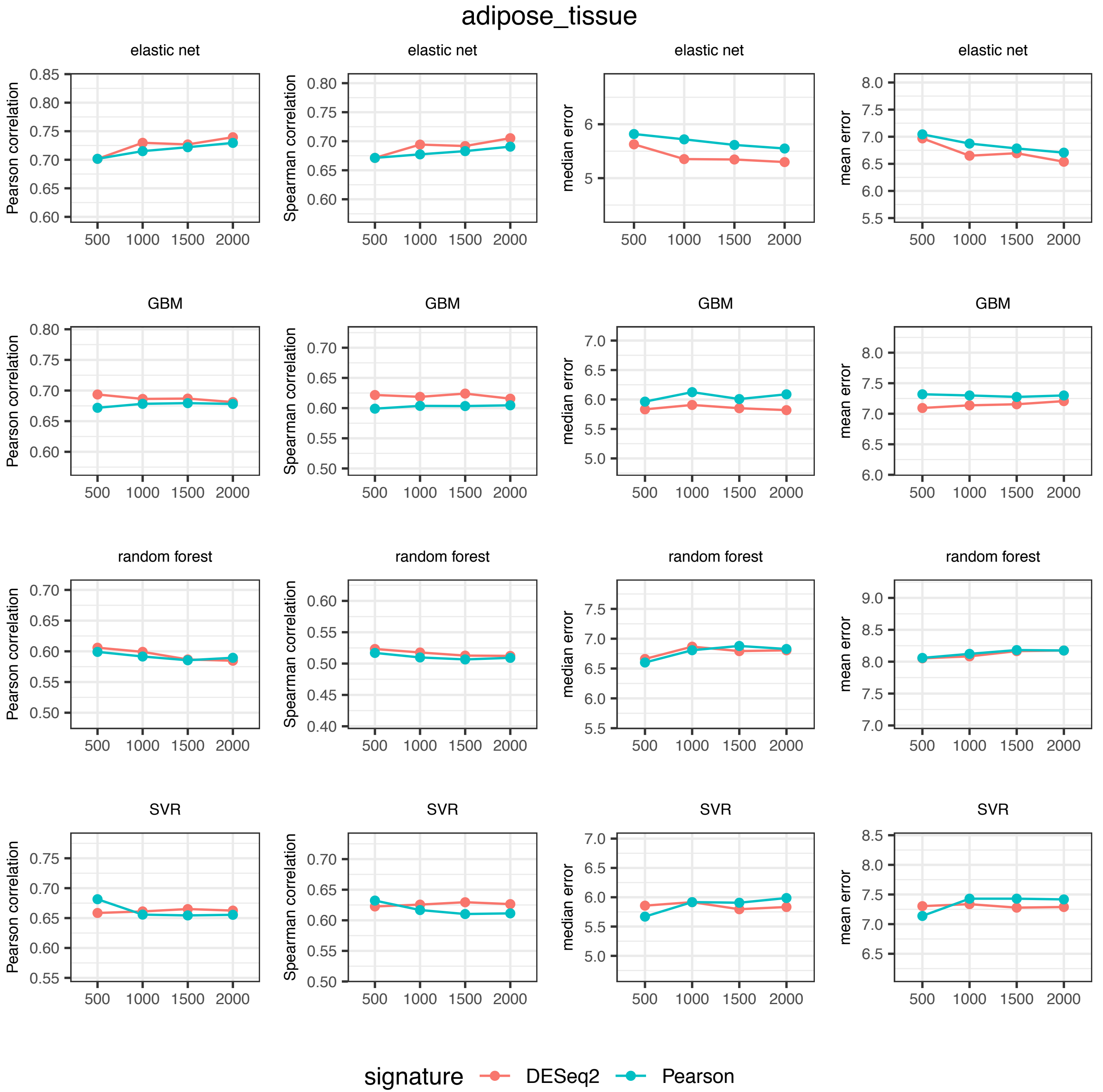

adipose_tissue
elastic net
GBM
random forest
SVR
Pearson correlation
Spearman correlation
median error
mean error
500
1000
1500
2000
signature
DESeq2
Pearson

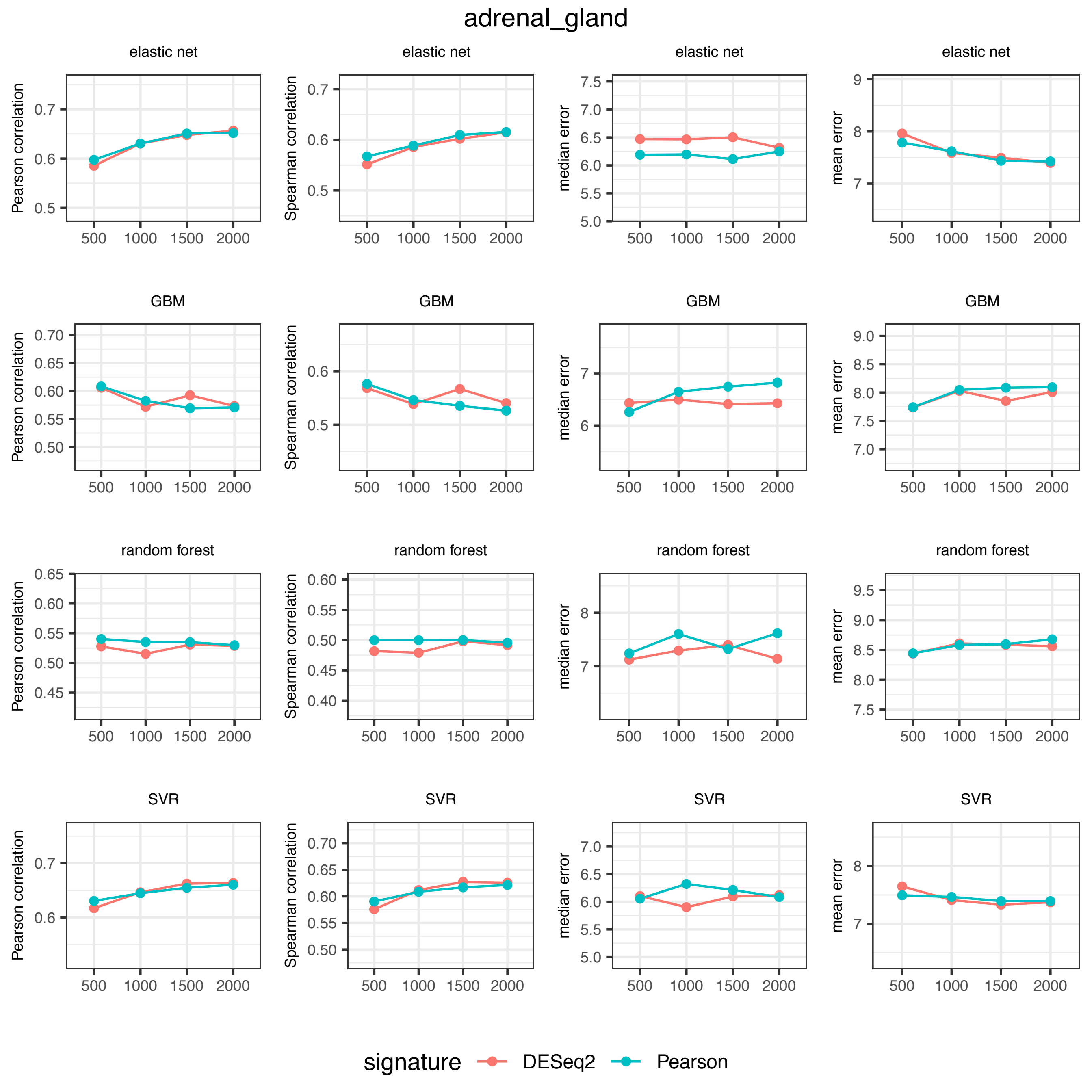
adrenal_gland
elastic net
GBM
random forest
SVR
Pearson correlation
Spearman correlation
median error
mean error
500
1000
1500
2000
signature
DESeq2
Pearson

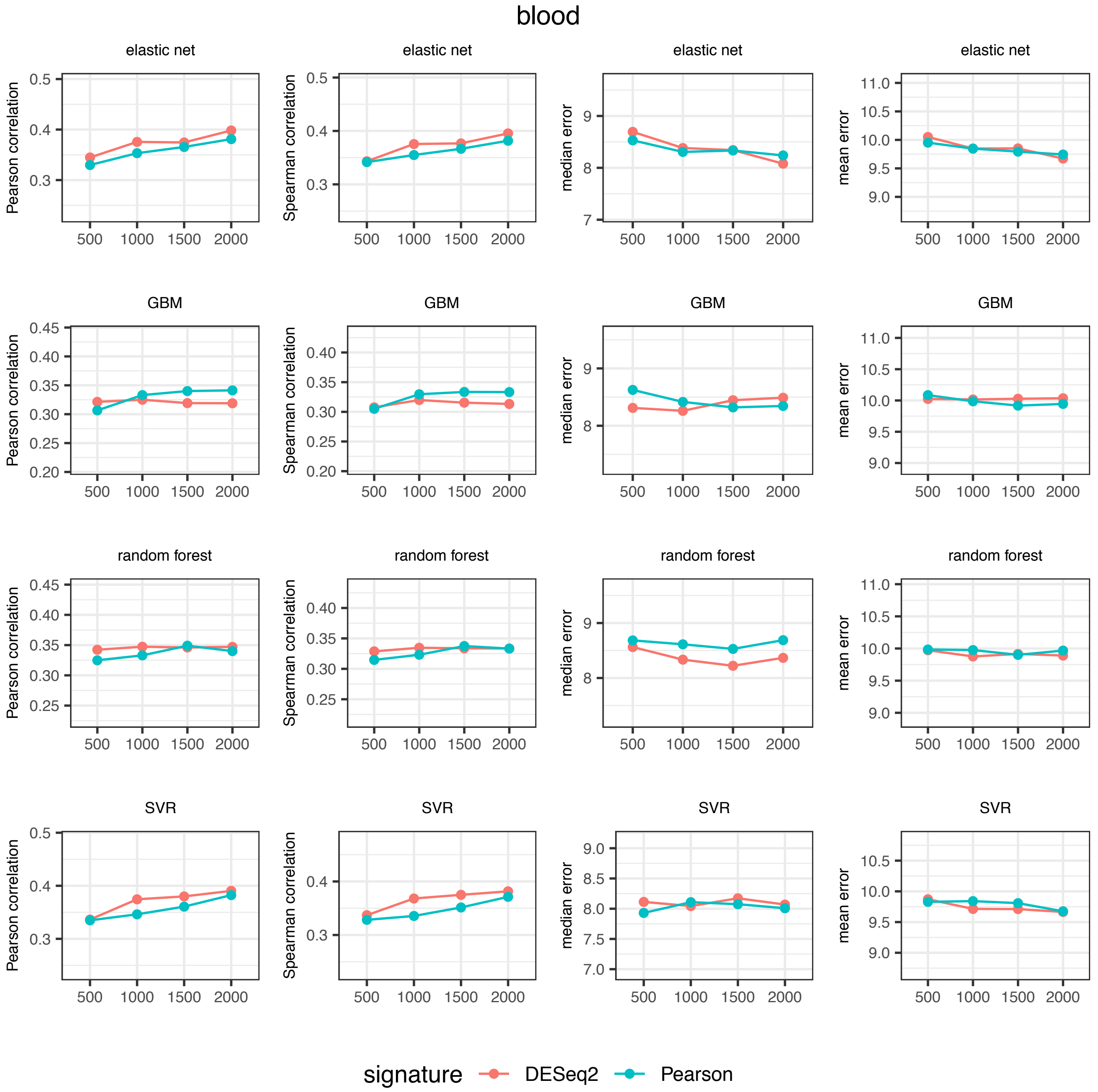
blood
elastic net
GBM
random forest
SVR
Pearson correlation
Spearman correlation
median error
mean error
500
1000
1500
2000
signature
DESeq2
Pearson

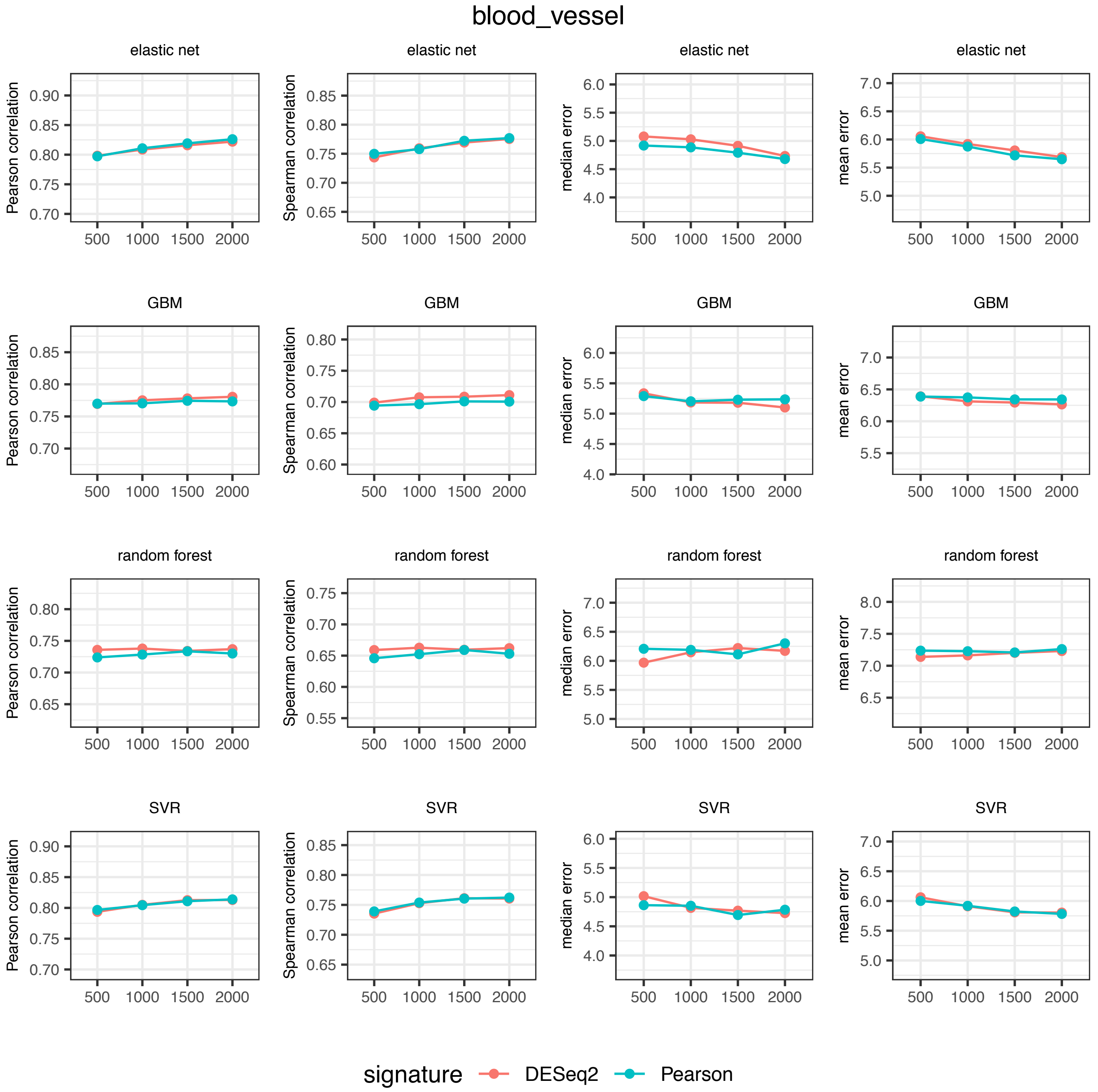
blood_vessel
elastic net
GBM
random forest
SVR
Pearson correlation
Spearman correlation
median error
mean error
500
1000
1500
2000
signature
DESeq2
Pearson

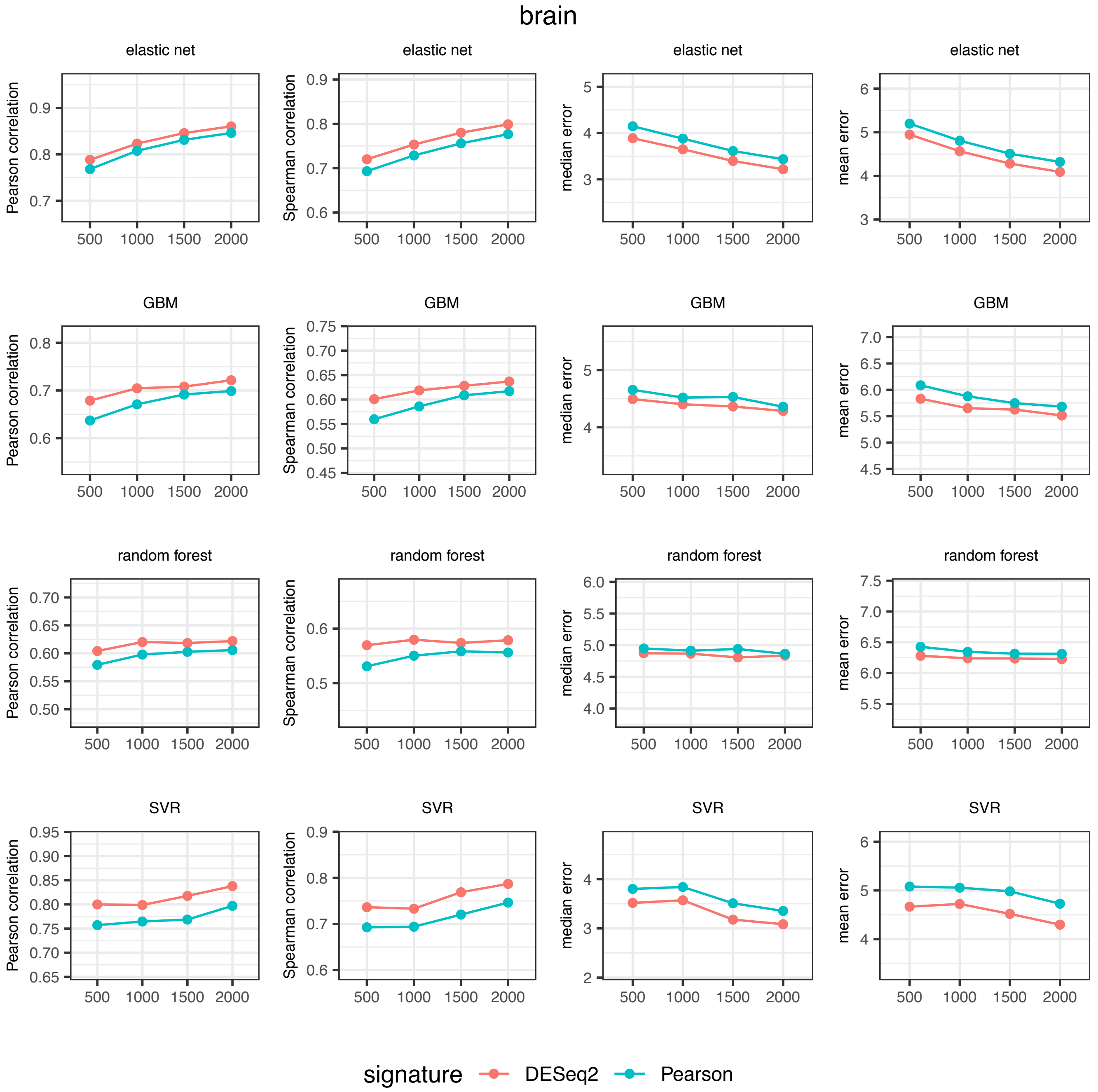

brain
elastic net
GBM
random forest
SVR
Pearson correlation
Spearman correlation
median error
mean error
500
1000
1500
2000
signature
DESeq2
Pearson

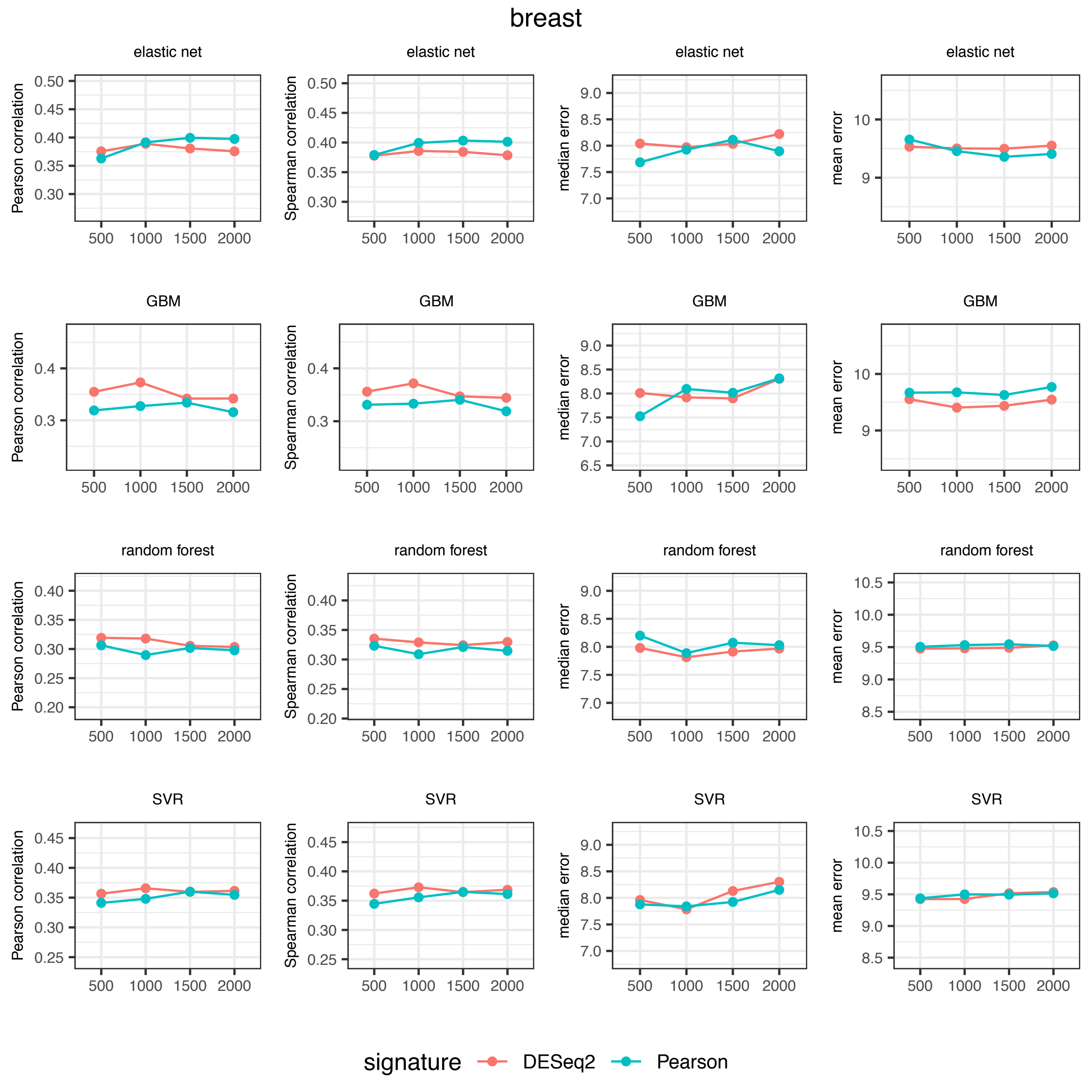
breast
elastic net
GBM
random forest
SVR
Pearson correlation
Spearman correlation
median error
mean error
500
1000
1500
2000
signature
DESeq2
Pearson

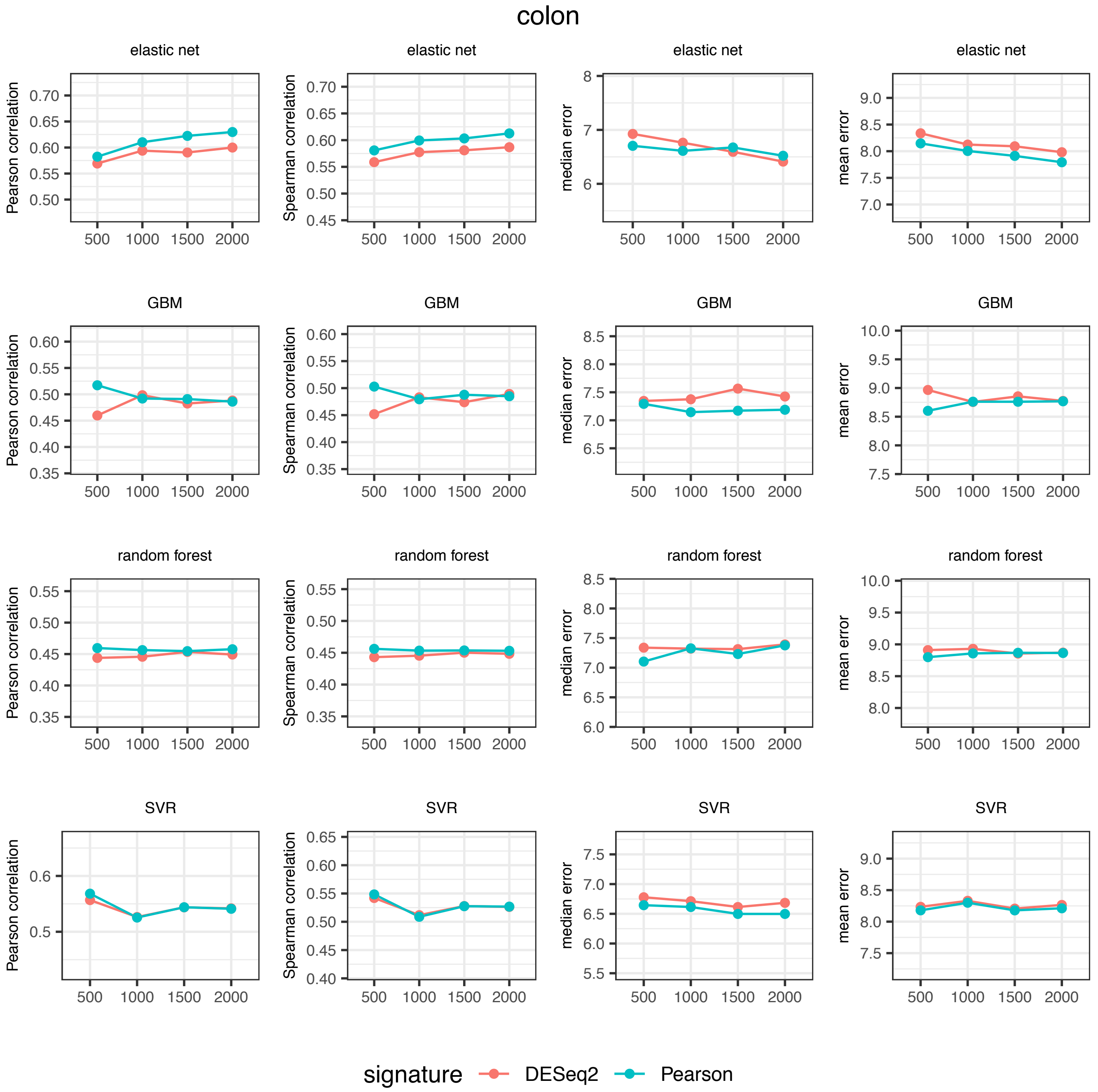

colon
elastic net
GBM
random forest
SVR
Pearson correlation
Spearman correlation
median error
mean error
500
1000
1500
2000
signature
DESeq2
Pearson

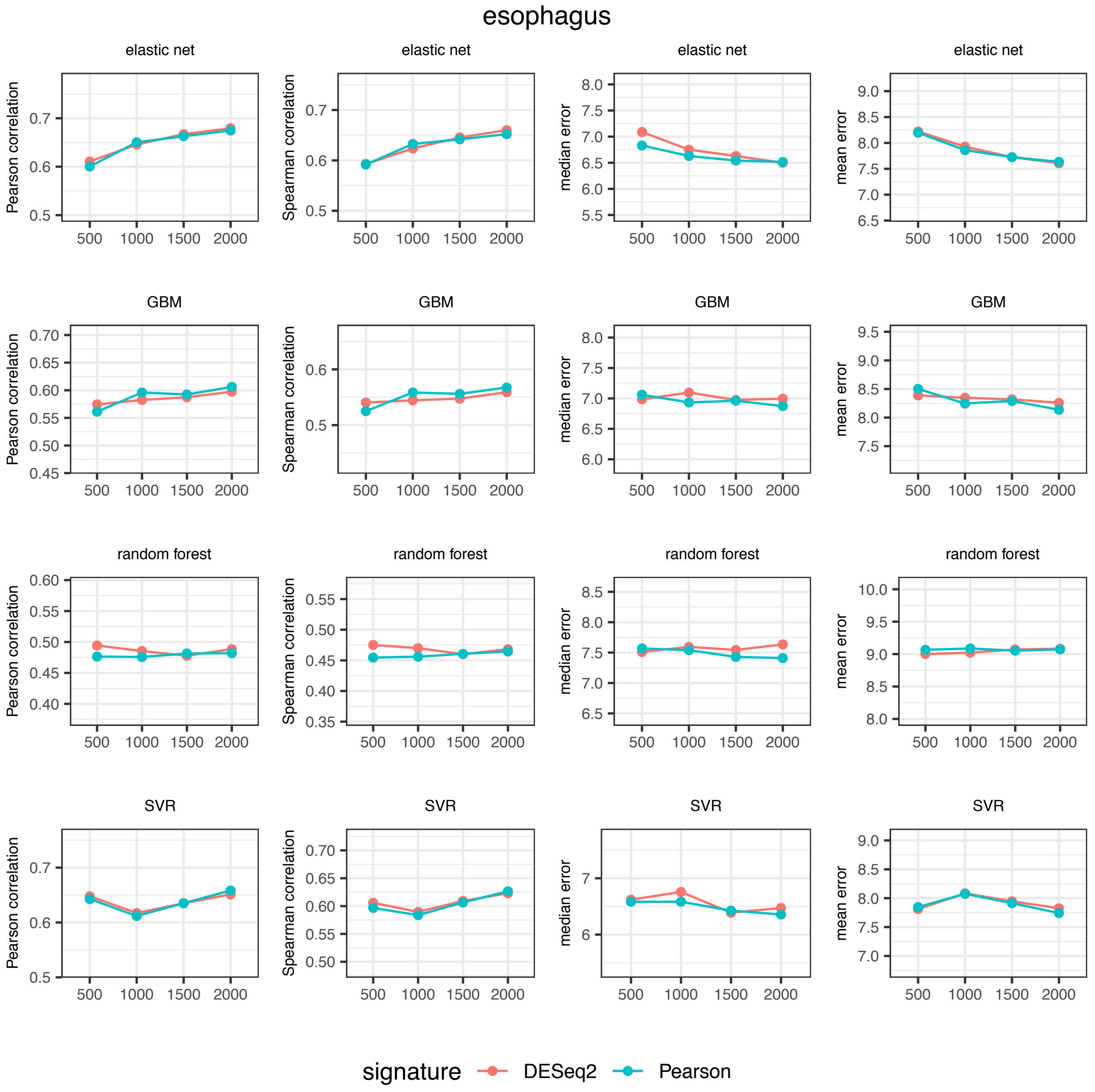

esophagus
elastic net
GBM
random forest
SVR
Pearson correlation
Spearman correlation
median error
mean error
500
1000
1500
2000
signature
DESeq2
Pearson

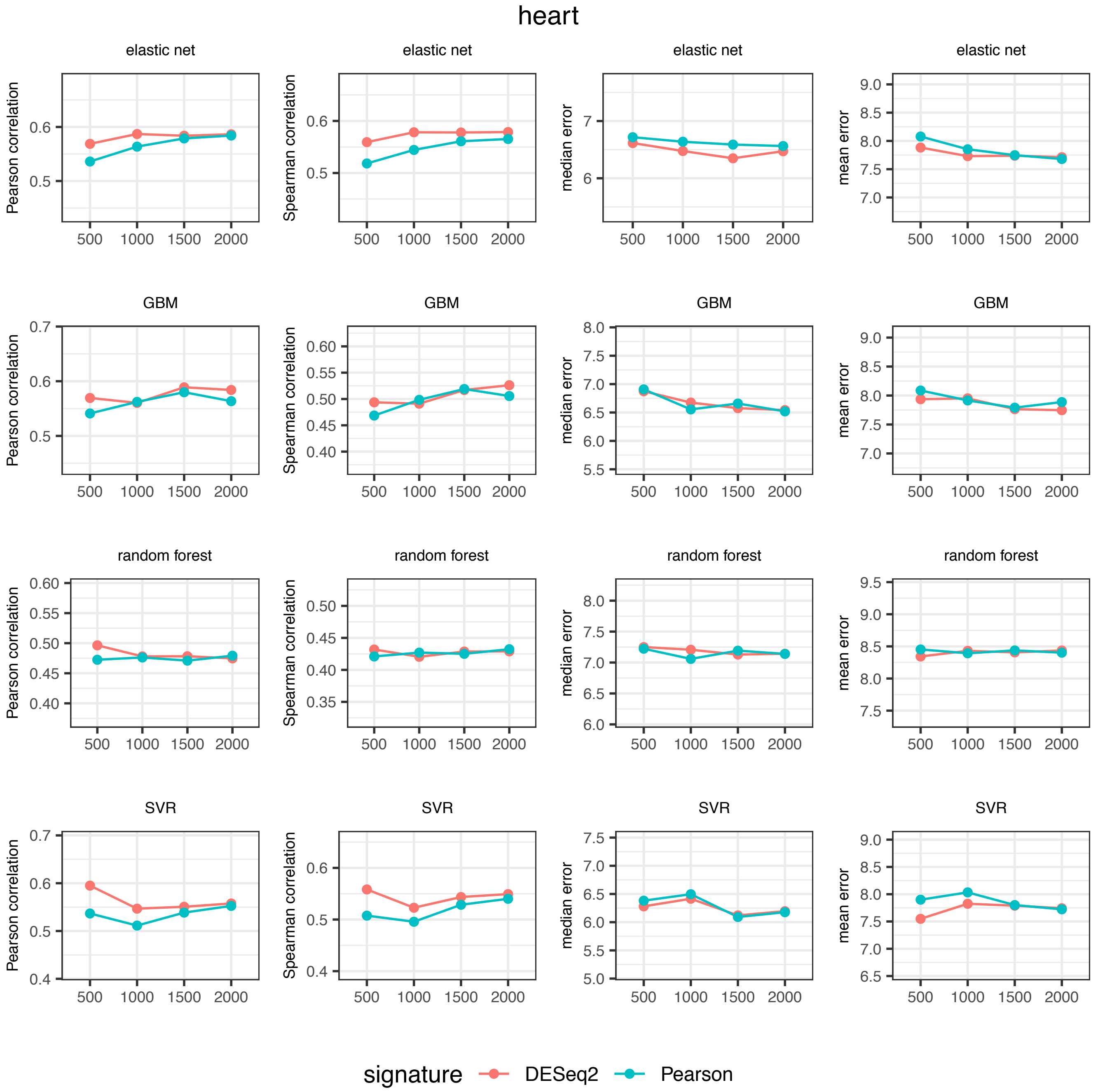
heart
elastic net
GBM
random forest
SVR
Pearson correlation
Spearman correlation
median error
mean error
500
1000
1500
2000
signature
DESeq2
Pearson

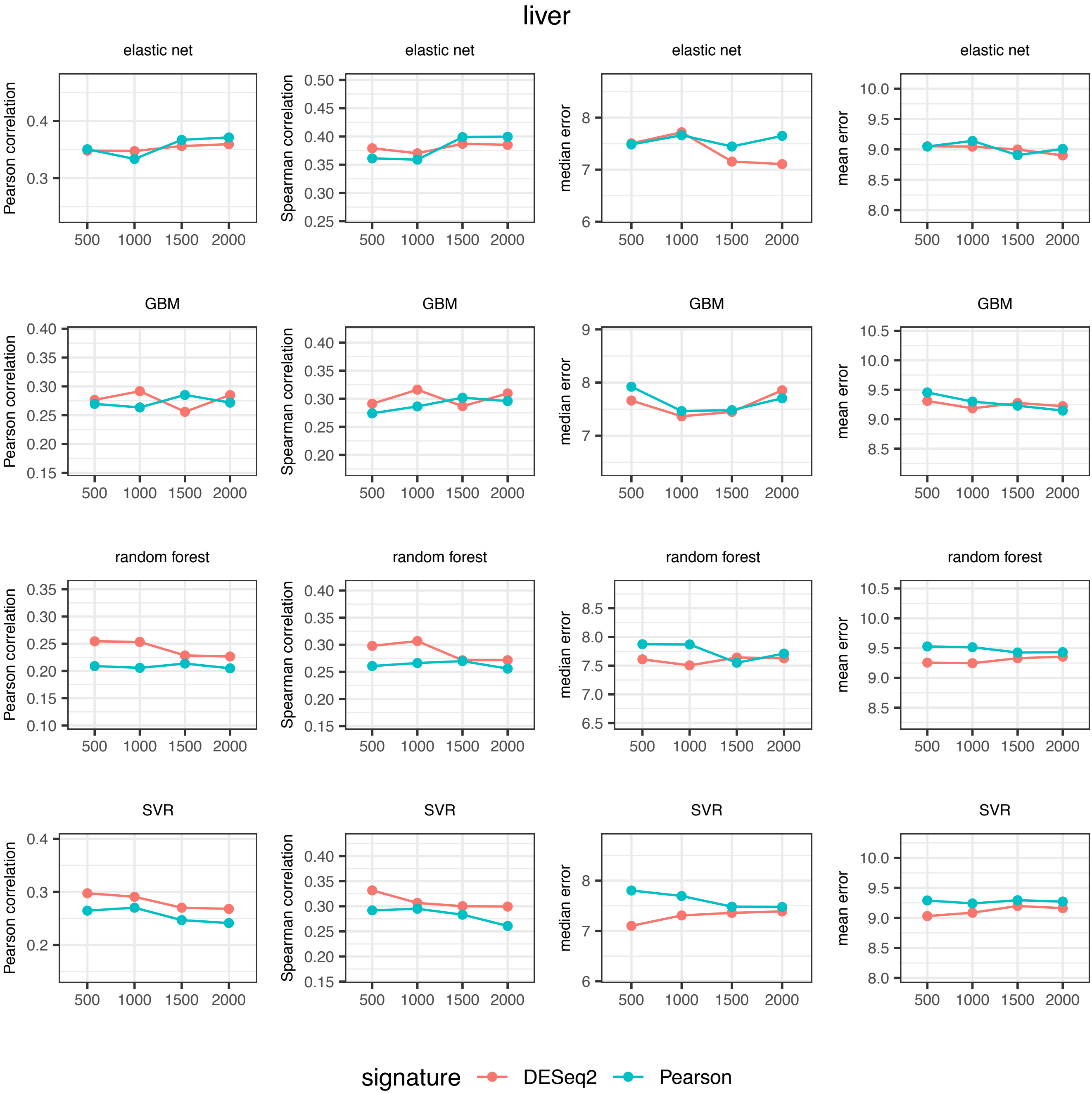
liver
elastic net
GBM
random forest
SVR
Pearson correlation
Spearman correlation
median error
mean error
500
1000
1500
2000
signature
DESeq2
Pearson

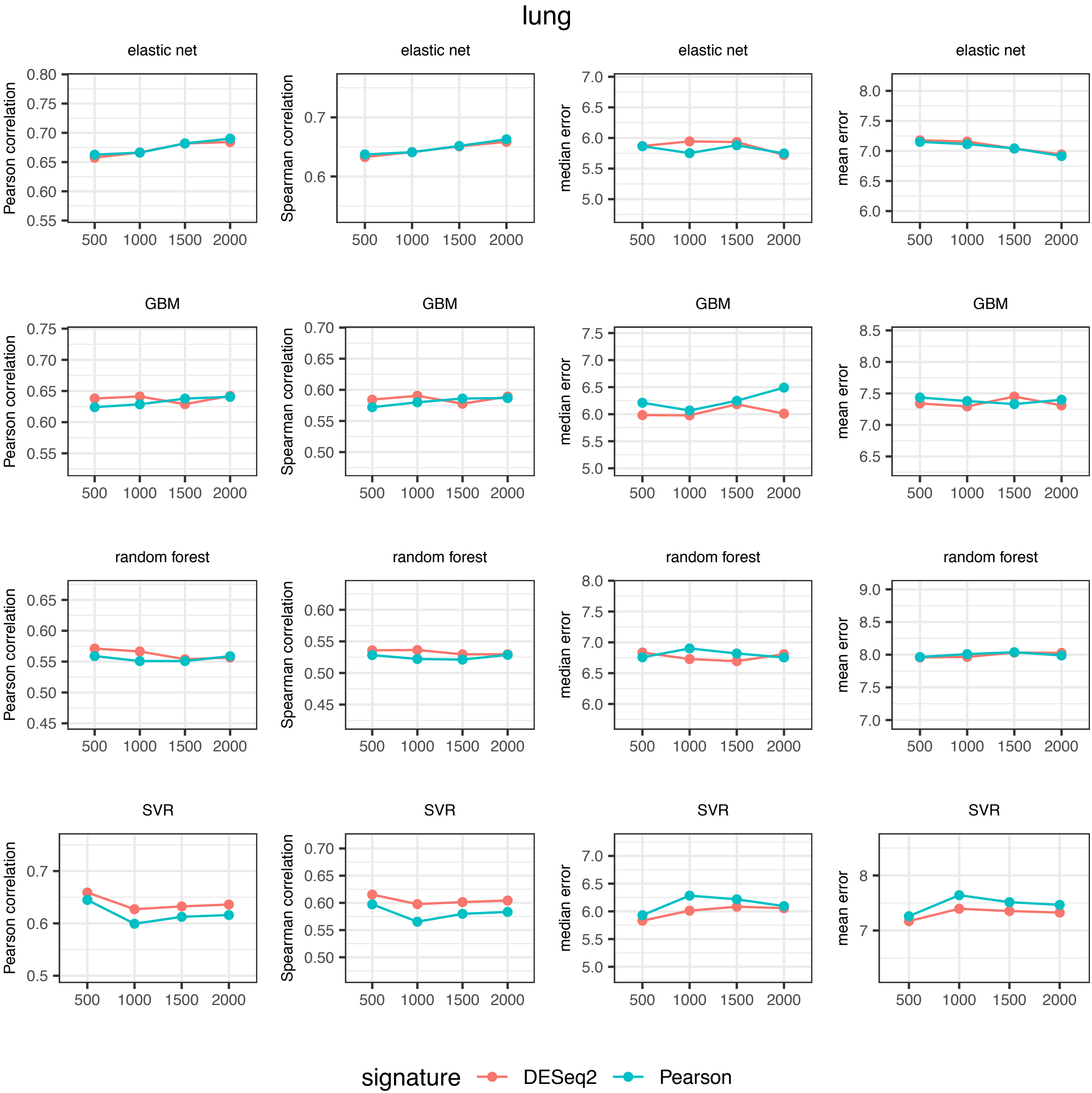

lung
elastic net
GBM
random forest
SVR
Pearson correlation
Spearman correlation
median error
mean error
500
1000
1500
2000
signature
DESeq2
Pearson

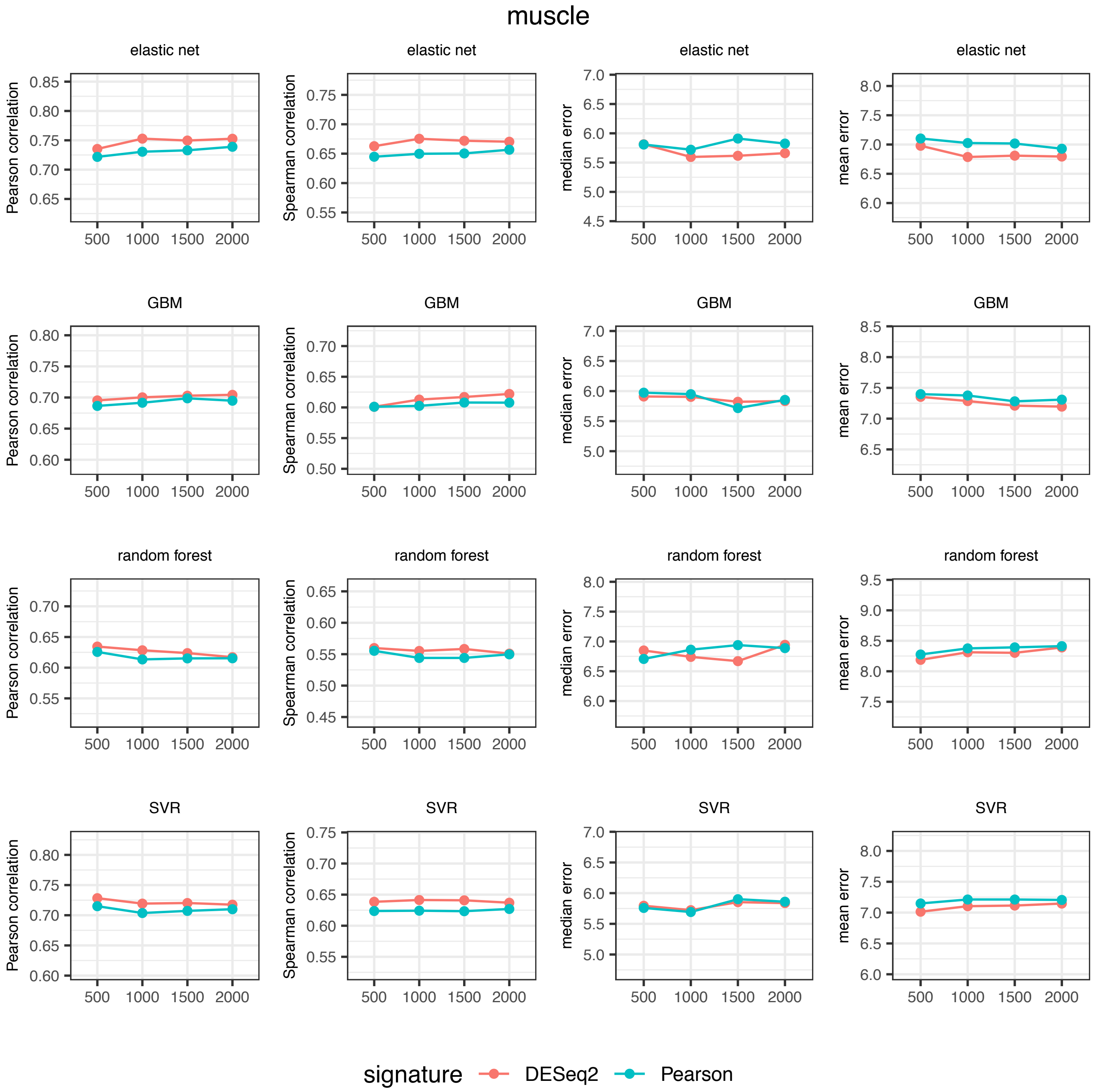
muscle
elastic net
GBM
random forest
SVR
Pearson correlation
Spearman correlation
median error
mean error
500
1000
1500
2000
signature
DESeq2
Pearson

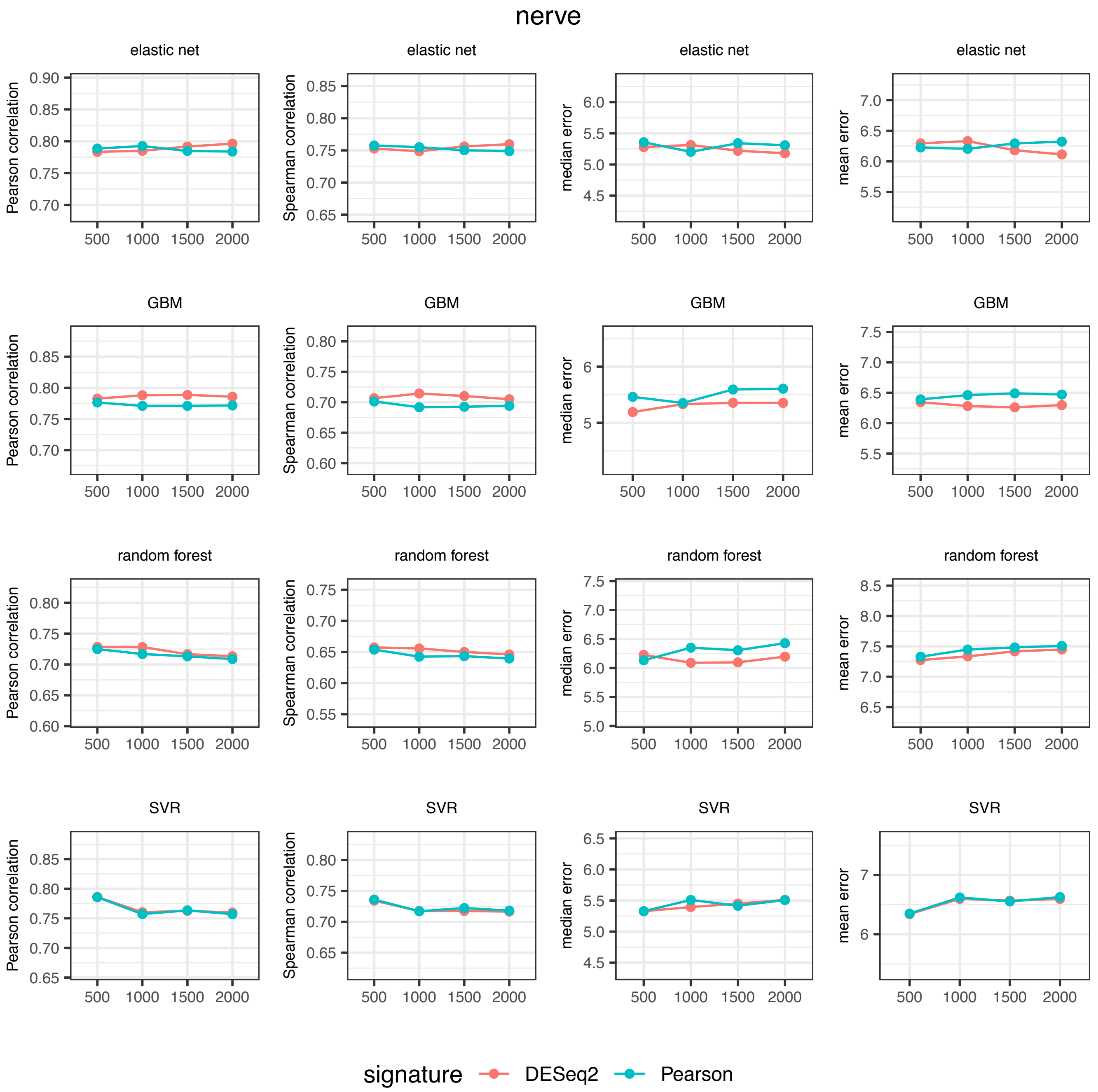
nerve
elastic net
GBM
random forest
SVR
Pearson correlation
Spearman correlation
median error
mean error
500
1000
1500
2000
signature
DESeq2
Pearson

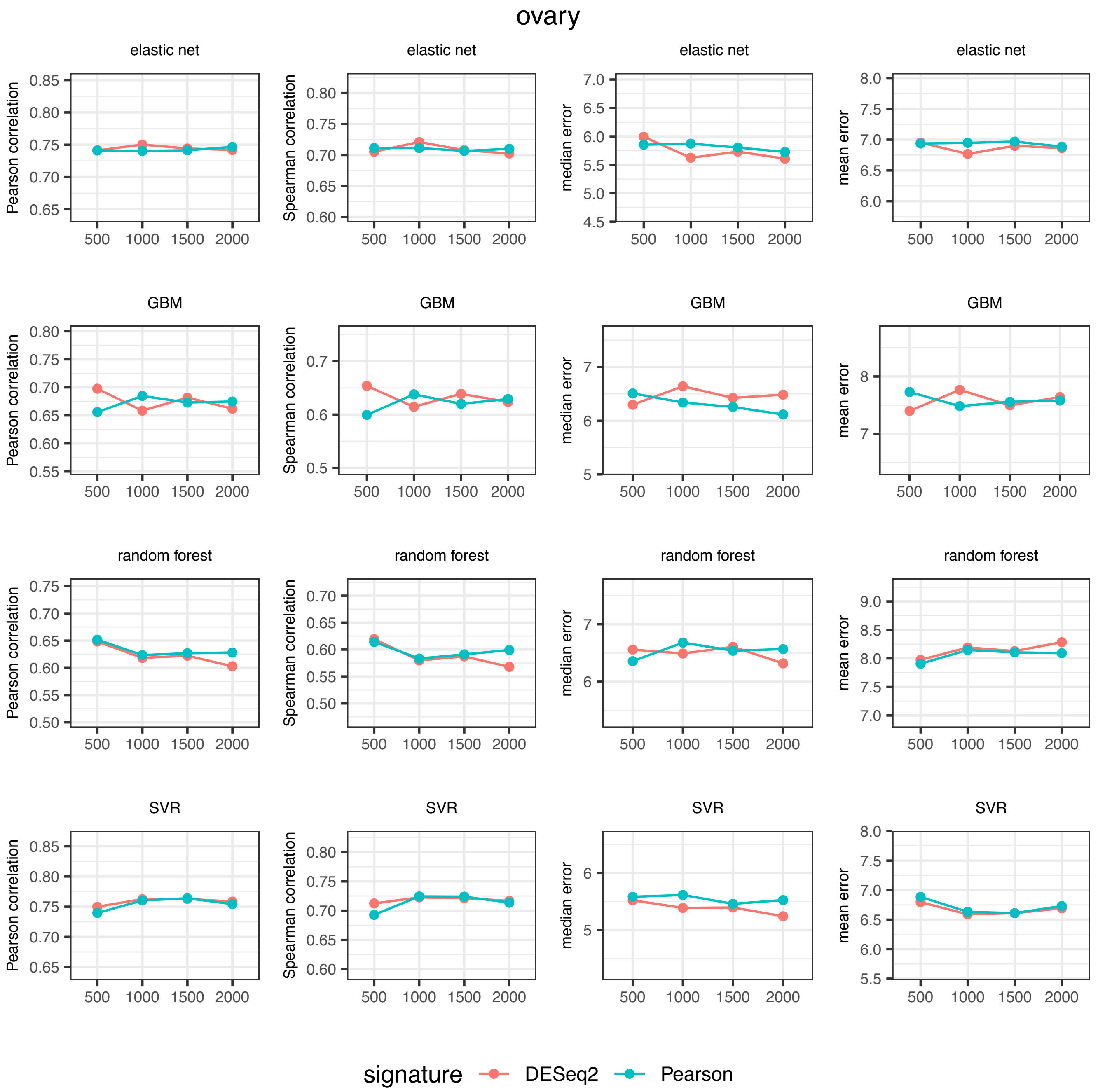

ovary
elastic net
GBM
random forest
SVR
Pearson correlation
Spearman correlation
median error
mean error
500
1000
1500
2000
signature
DESeq2
Pearson

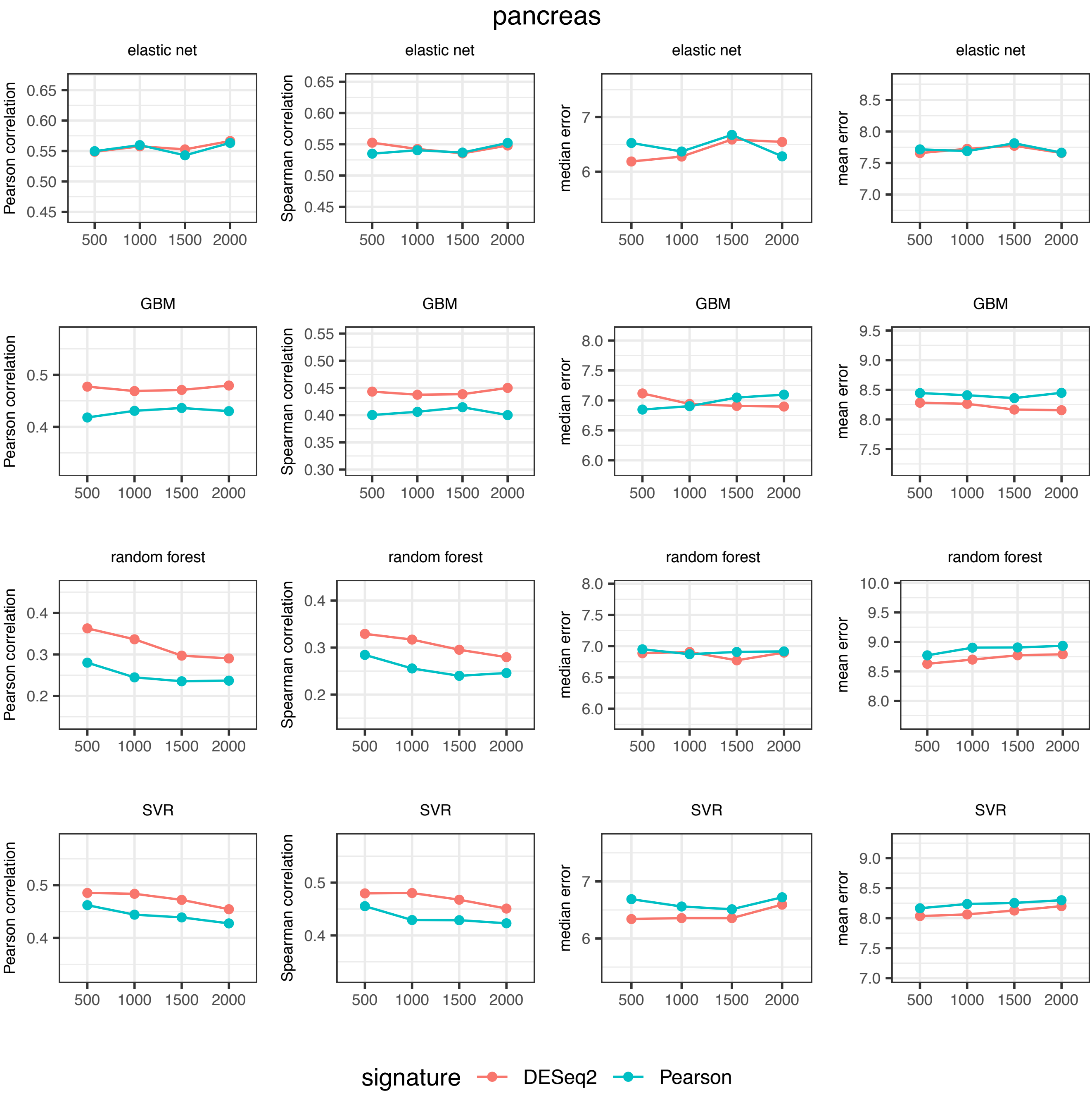
pancreas
elastic net
GBM
random forest
SVR
Pearson correlation
Spearman correlation
median error
mean error
500
1000
1500
2000
signature
DESeq2
Pearson

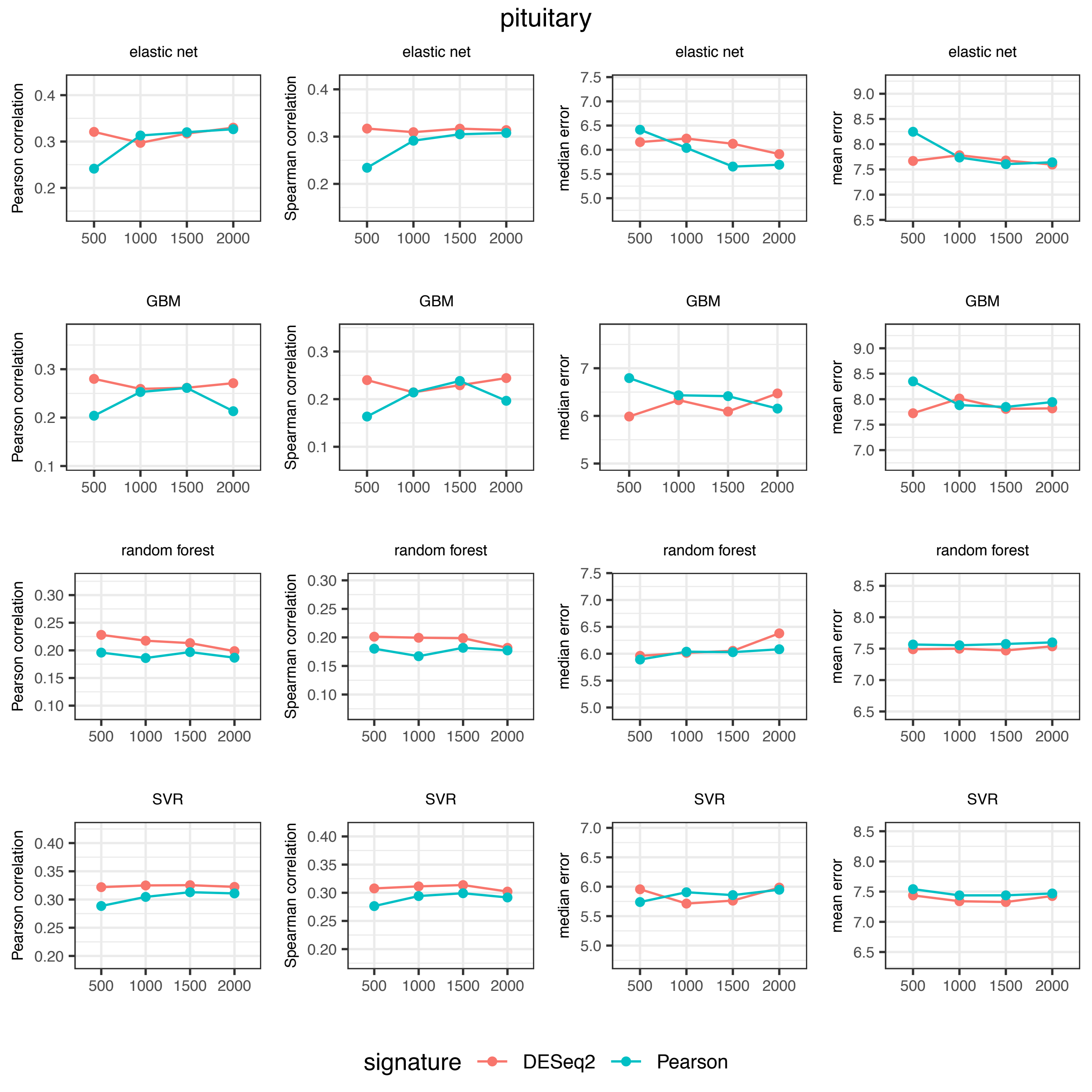
pituitary
elastic net
GBM
random forest
SVR
Pearson correlation
Spearman correlation
median error
mean error
500
1000
1500
2000
signature
DESeq2
Pearson

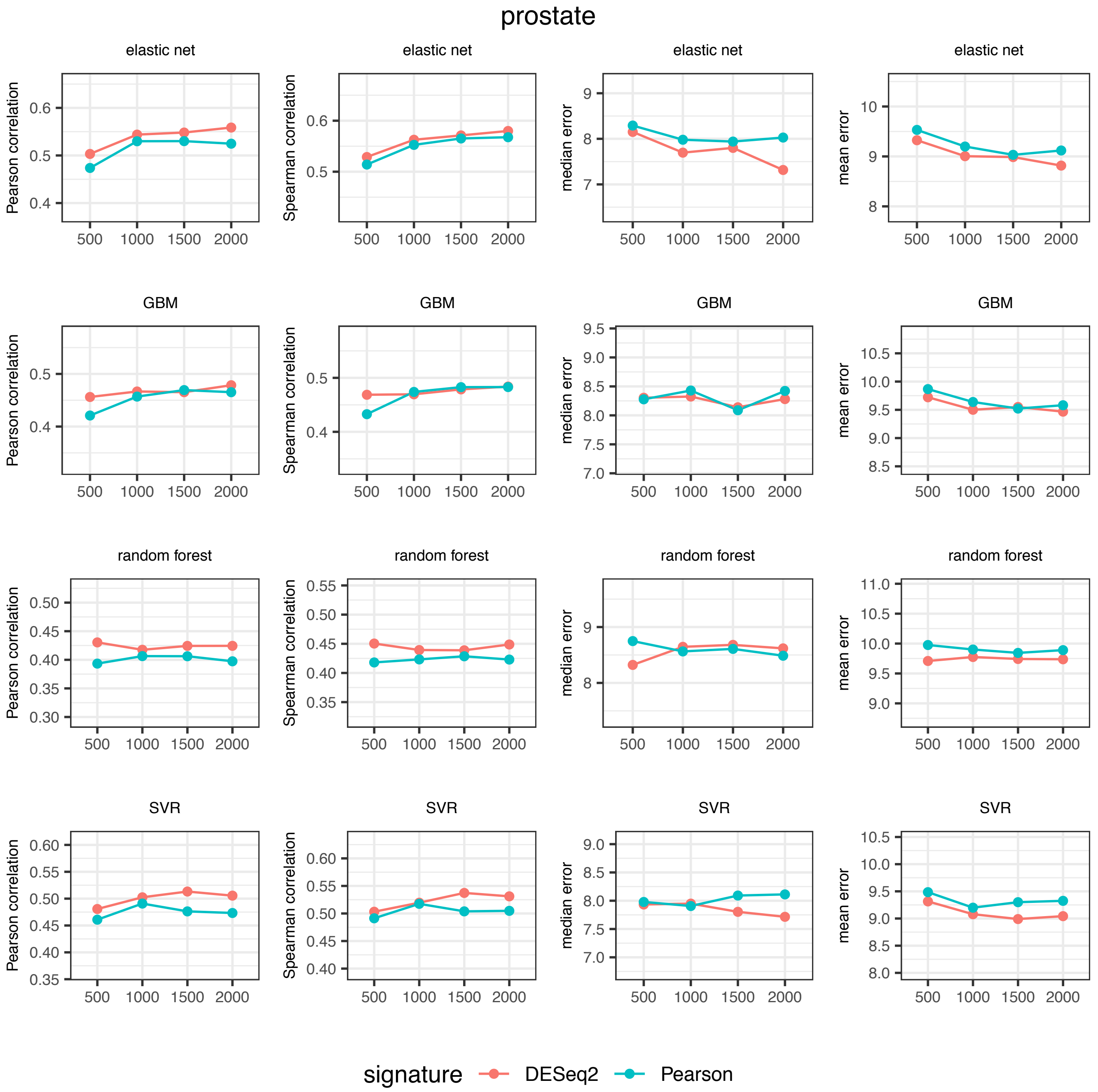

prostate
elastic net
GBM
random forest
SVR
Pearson correlation
Spearman correlation
median error
mean error
500
1000
1500
2000
signature
DESeq2
Pearson

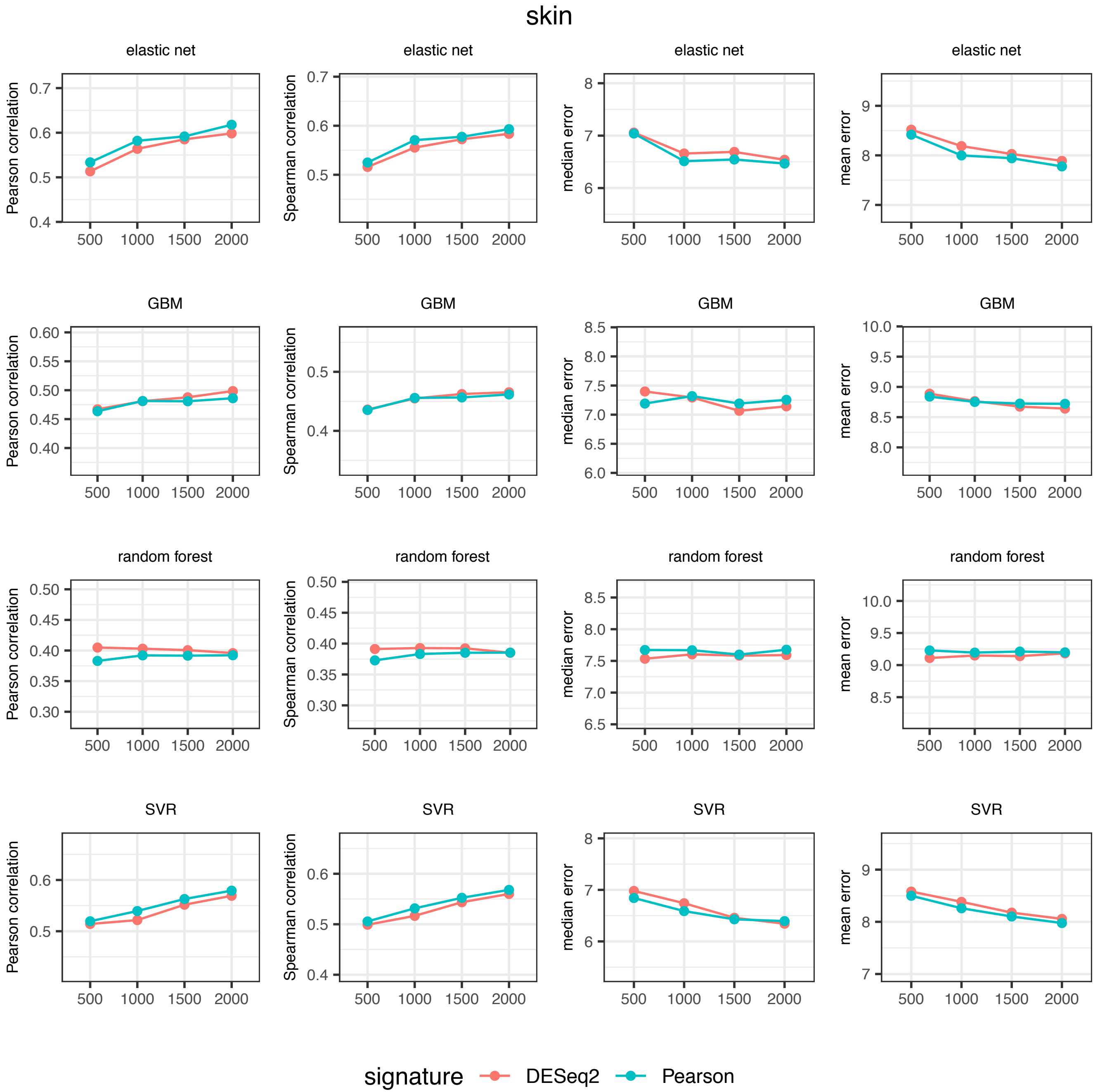

skin
elastic net
GBM
random forest
SVR
Pearson correlation
Spearman correlation
median error
mean error
500
1000
1500
2000
signature
DESeq2
Pearson

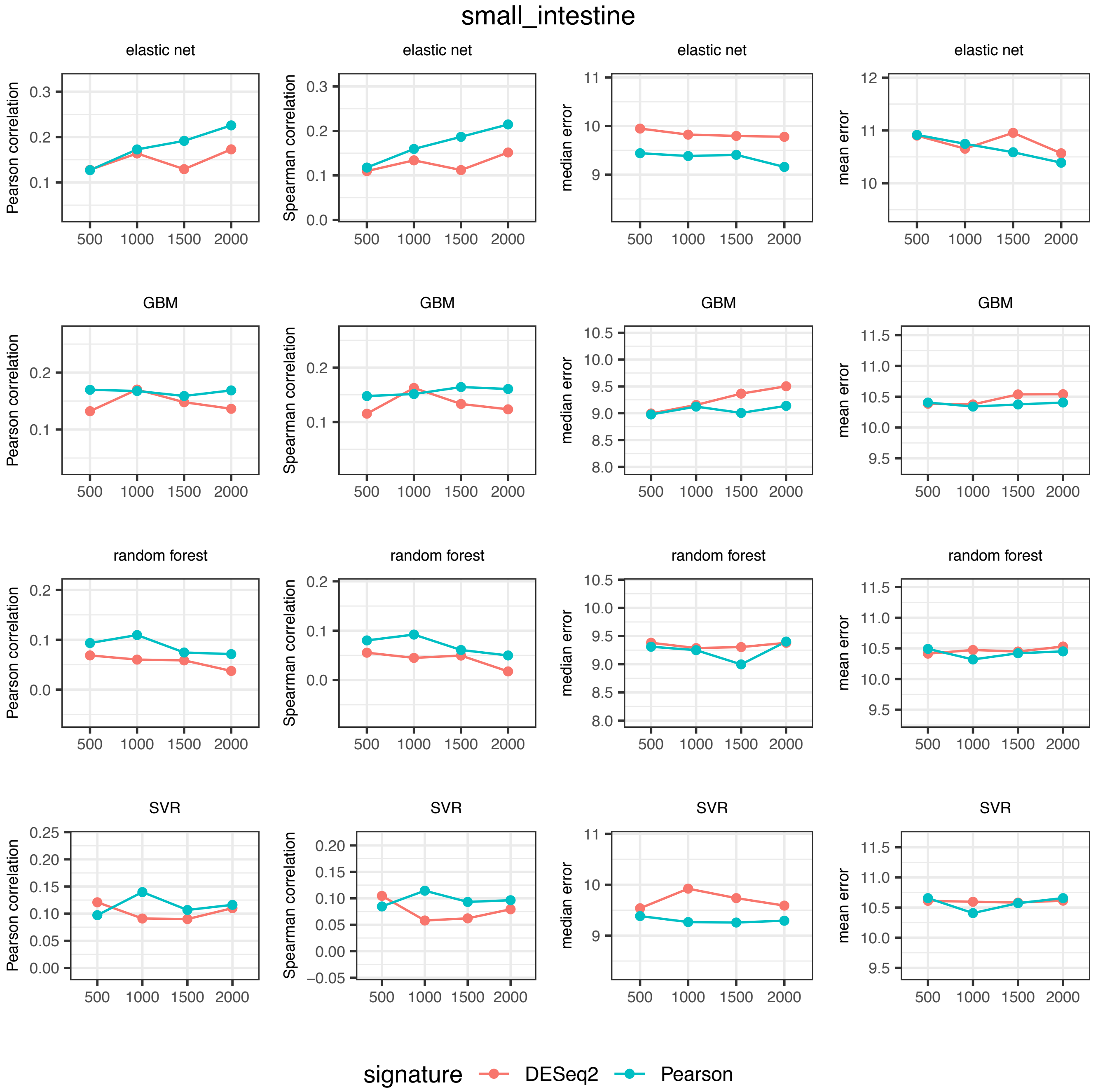
small_intestine
elastic net
GBM
random forest
SVR
Pearson correlation
Spearman correlation
median error
mean error
500
1000
1500
2000
signature
DESeq2
Pearson

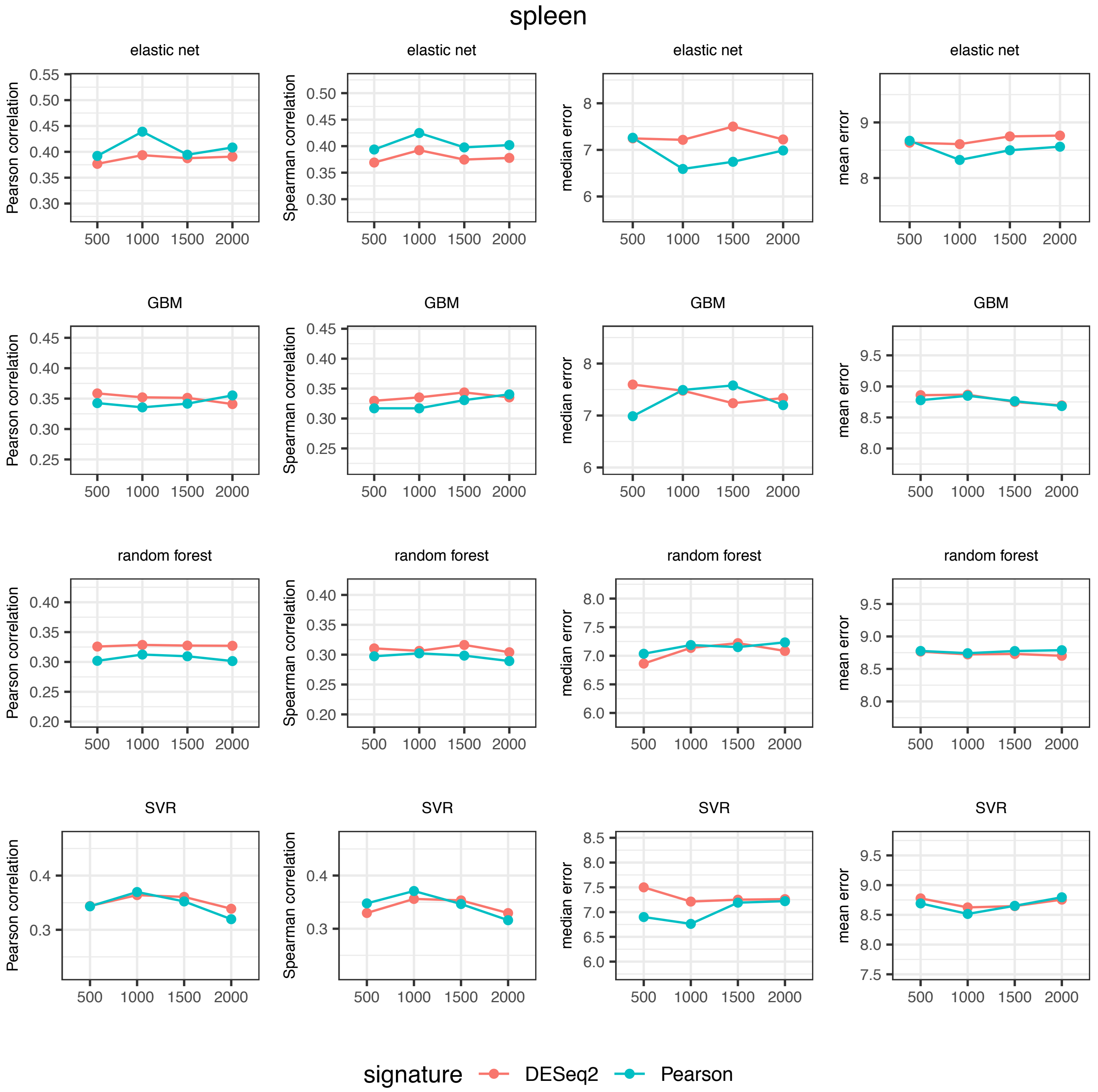
spleen
elastic net
GBM
random forest
SVR
Pearson correlation
Spearman correlation
median error
mean error
500
1000
1500
2000
signature
DESeq2
Pearson

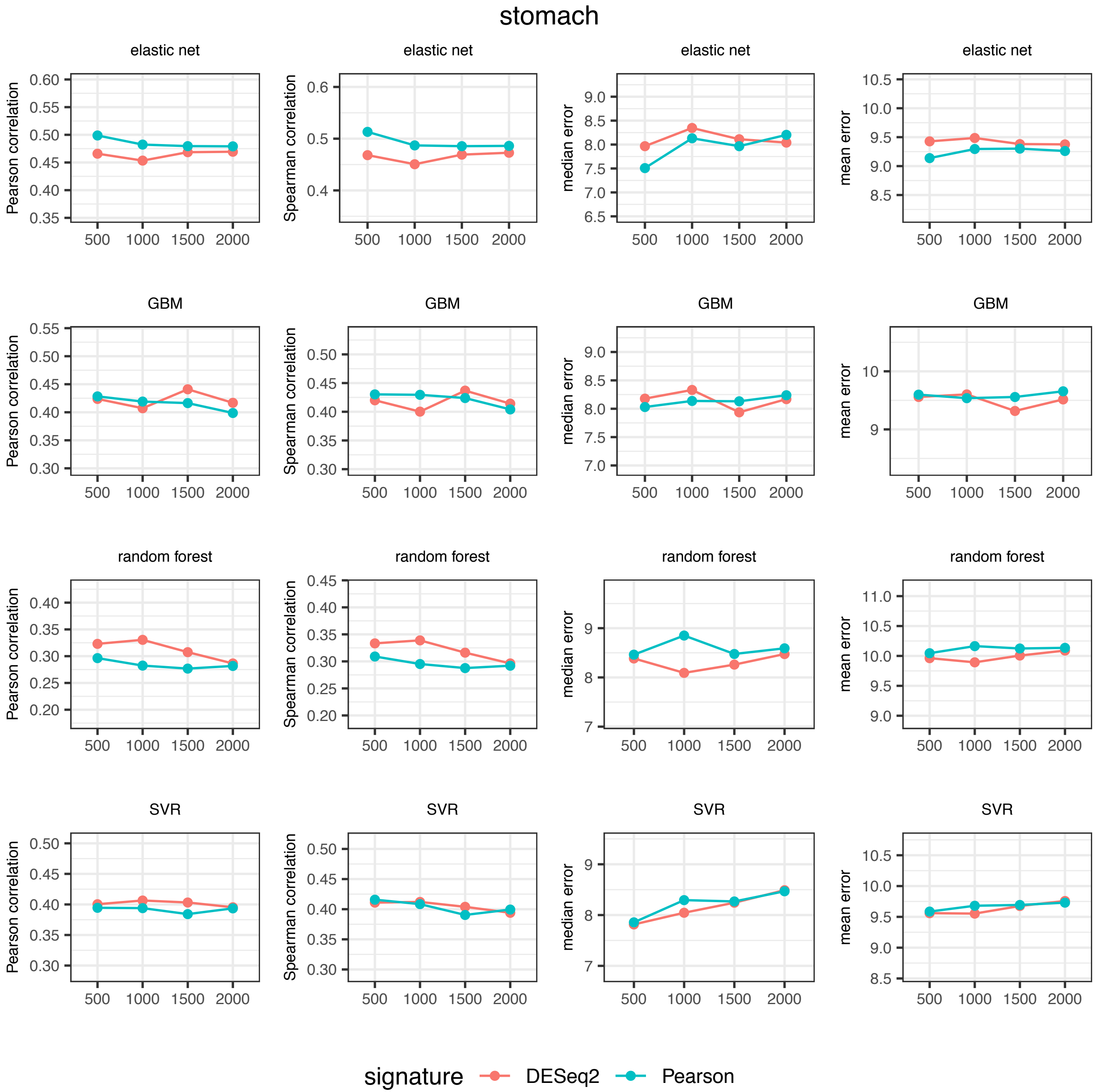
stomach
elastic net
GBM
random forest
SVR
Pearson correlation
Spearman correlation
median error
mean error
500
1000
1500
2000
signature
DESeq2
Pearson

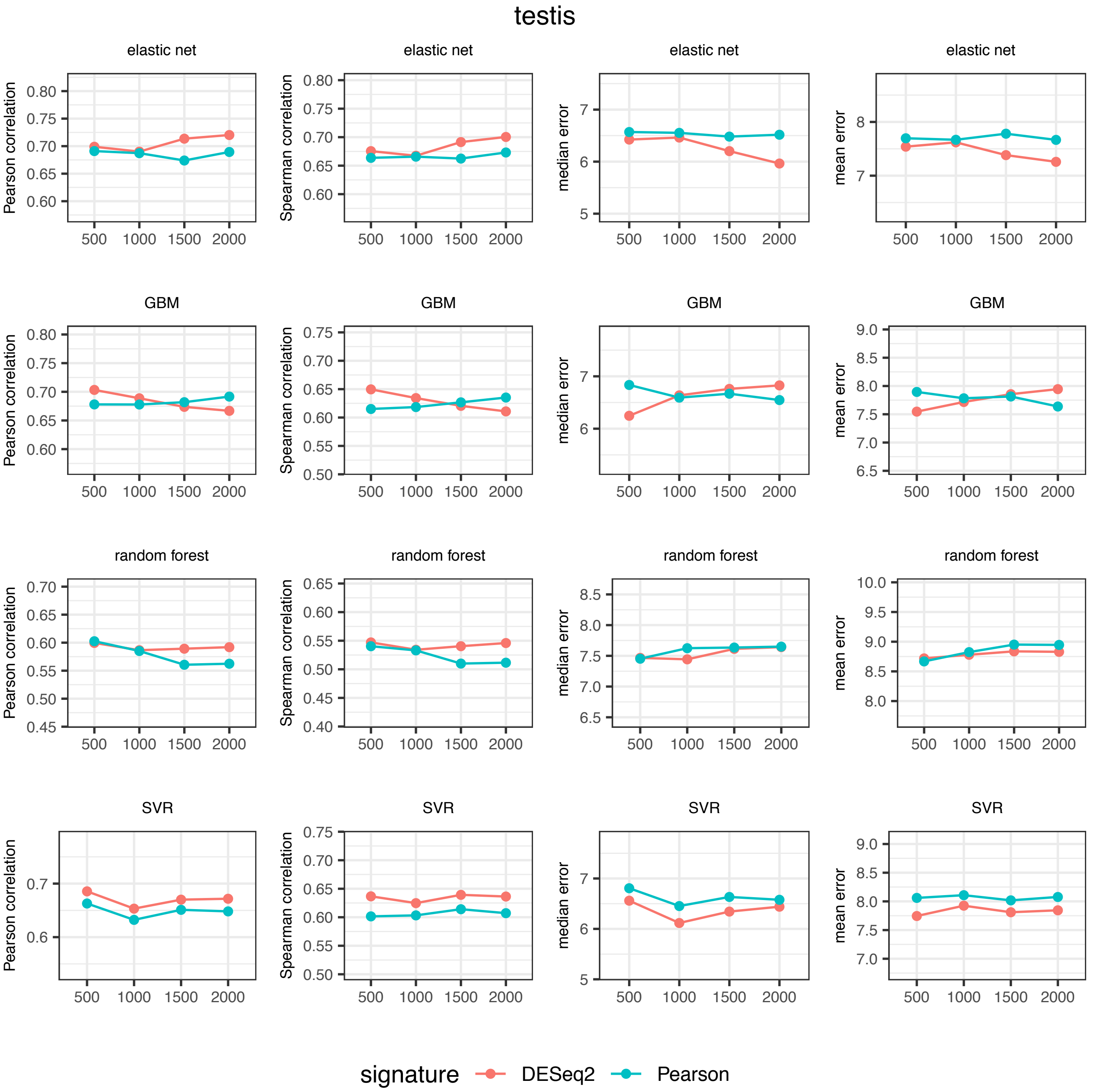

testis
elastic net
GBM
random forest
SVR
Pearson correlation
Spearman correlation
median error
mean error
500
1000
1500
2000
signature
DESeq2
Pearson

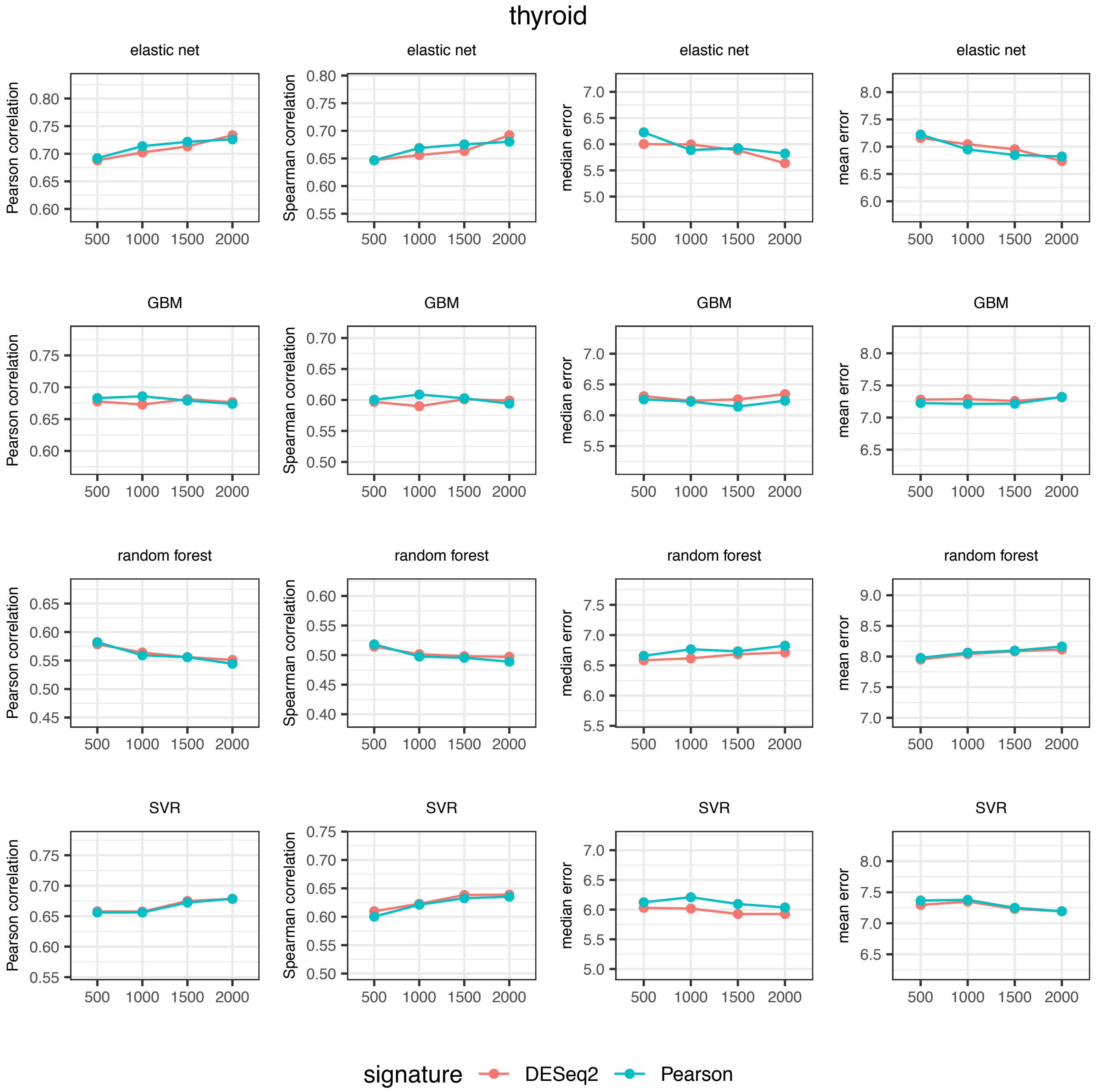
thyroid
elastic net
GBM
random forest
SVR
Pearson correlation
Spearman correlation
median error
mean error
500
1000
1500
2000
signature
DESeq2
Pearson

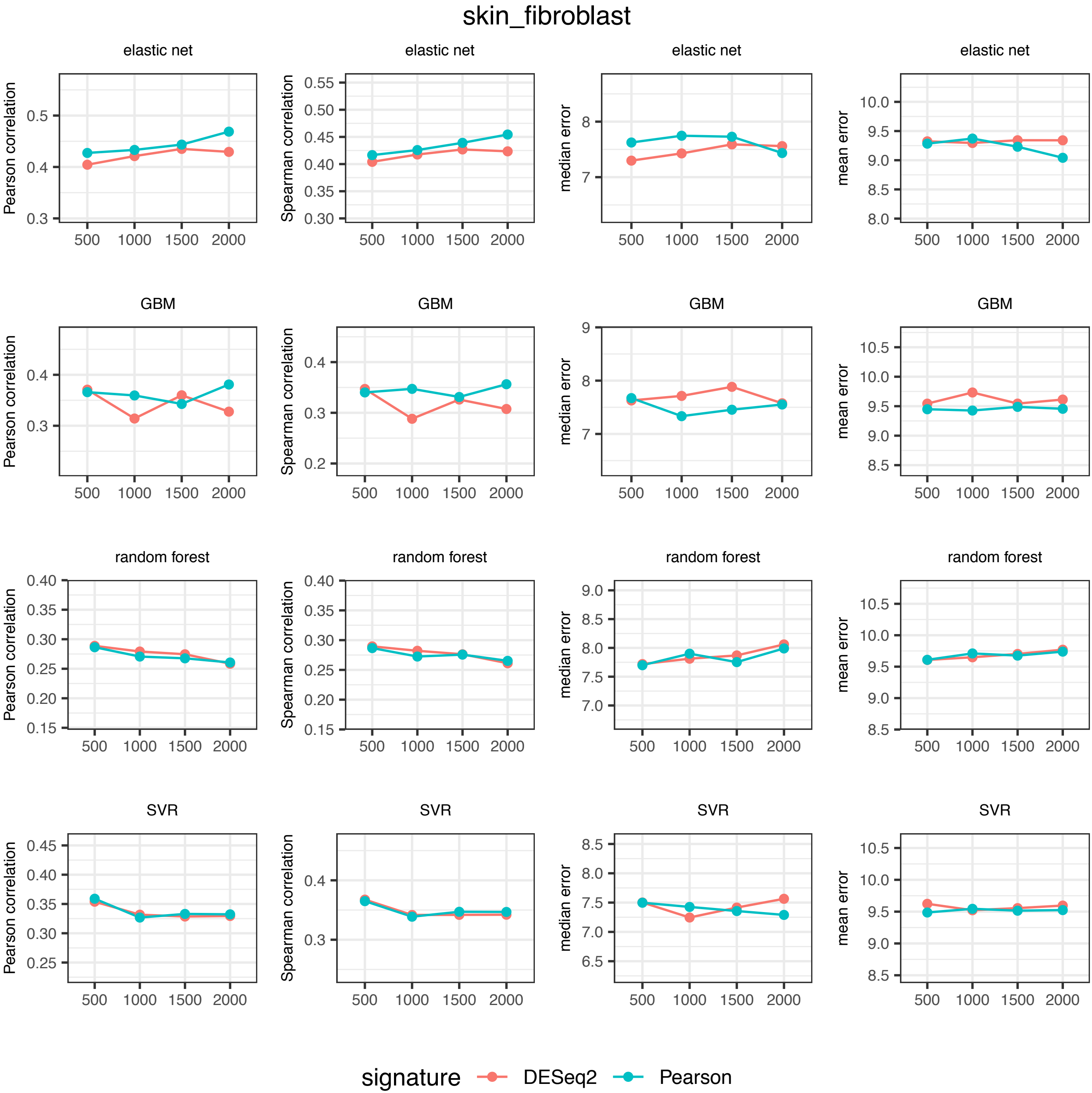
skin_fibroblast
elastic net
GBM
random forest
SVR
Pearson correlation
Spearman correlation
median error
mean error
500
1000
1500
2000
signature
DESeq2
Pearson

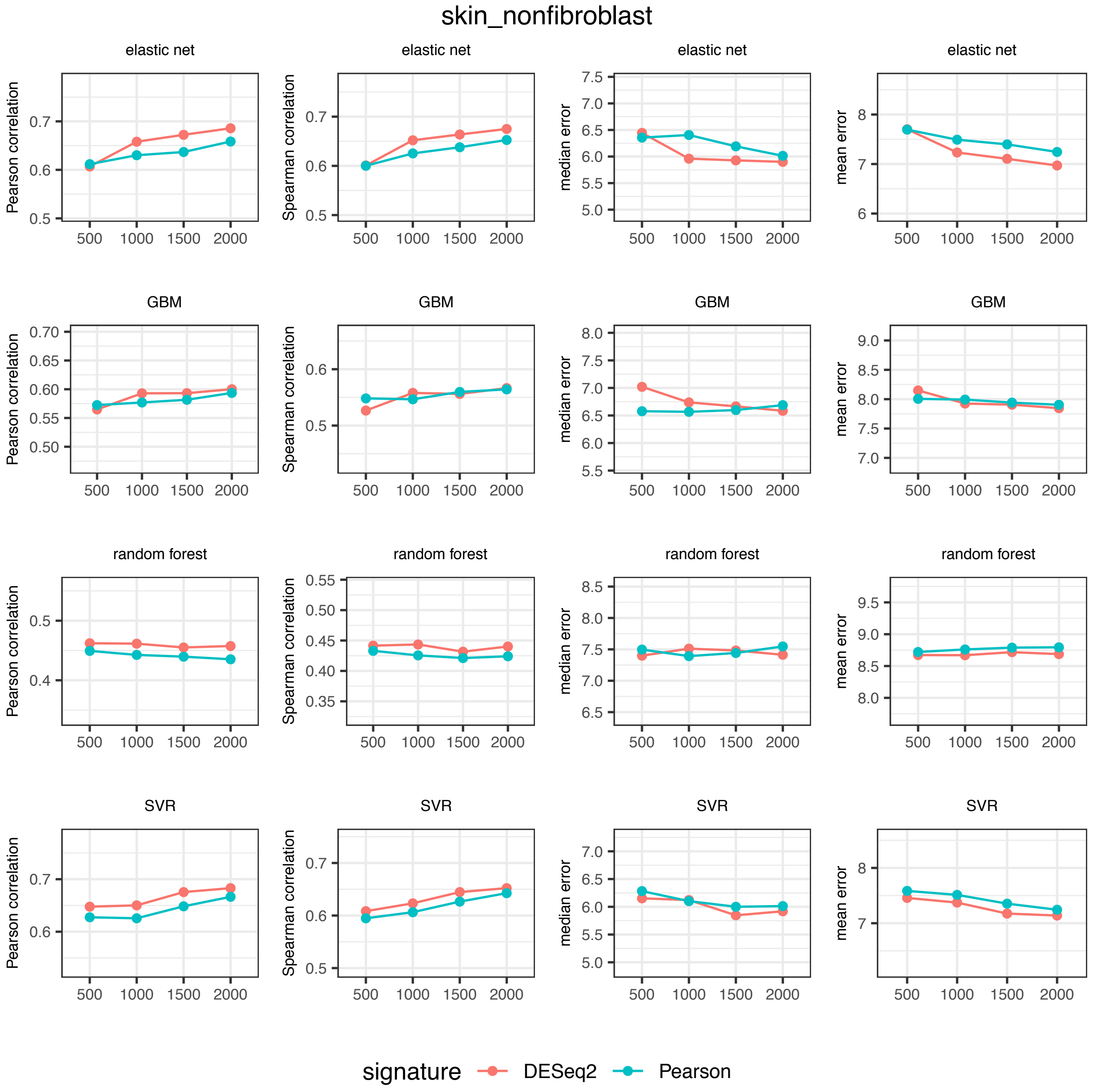
skin_nonfibroblast
elastic net
GBM
random forest
SVR
Pearson correlation
Spearman correlation
median error
mean error
500
1000
1500
2000
signature
DESeq2
Pearson
